# Gut microbiota analyses of inflammatory bowel diseases from a representative Saudi population

**DOI:** 10.1186/s12876-023-02904-2

**Published:** 2023-07-28

**Authors:** Raed M. Alsulaiman, Abdulaziz A. Al-Quorain, Fahad A. Al-Muhanna, Stanley Piotrowski, Ezzeddin A. Kurdi, Chittibabu Vatte, Ahmed A. Alquorain, Noorah H. Alfaraj, Abdulaziz M. Alrezuk, Fred Robinson, Alexa K. Dowdell, Turki A. Alamri, Lauren Hamilton, Hetal Lad, Hui Gao, Divya Gandla, Brendan J. Keating, Ryan Meng, Brian Piening, Amein K. Al-Ali

**Affiliations:** 1 Department of Internal Medicine, King Fahd Hospital of the University, Alkhobar, Imam Abdulrahman bin Faisal University, Dammam, 31441 Saudi Arabia; 2 Earle A Chiles Research Institute, Robert W. Franz Cancer Center, Portland, Oregon, OR 97213 USA; 3 King Fahd Hospital, Al Hafof, 36441 Saudi Arabia; 4 Department of Clinical Biochemistry, College of Medicine, Imam Abdulrahman bin Faisal University, Dammam, Saudi Arabia; 5 Al Qatif Central Hospital, Al Qatif, 32654 Saudi Arabia; 6 Department of Surgery, Perelman School of Medicine, University of Pennsylvania, Pennsylvania, PA 19104 USA

**Keywords:** IBD, Crohn’s disease, Ulcerative colitis, Inflammation, Microbiota, Saudi

## Abstract

**Background:**

Crohn’s diseases and ulcerative colitis, both of which are chronic immune-mediated disorders of the gastrointestinal tract are major contributors to the overarching Inflammatory bowel diseases. It has become increasingly evident that the pathological processes of IBDs results from interactions between genetic and environmental factors, which can skew immune responses against normal intestinal flora.

**Methods:**

The aim of this study is to assess and analyze the taxa diversity and relative abundances in CD and UC in the Saudi population. We utilized a sequencing strategy that targets all variable regions in the 16 S rRNA gene using the Swift Amplicon 16 S rRNA Panel on Illumina NovaSeq 6000.

**Results:**

The composition of stool 16 S rRNA was analyzed from 219 patients with inflammatory bowel disease and from 124 healthy controls. We quantified the abundance of microbial communities to examine any significant differences between subpopulations of samples. At the genus level, two genera in particular, *Veillonella* and *Lachnoclostridium* showed significant association with CD versus controls. There were significant differences between subjects with CD versus UC, with the top differential genera spanning *Akkermansia, Harryflintia, Maegamonas* and *Phascolarctobacterium*. Furthermore, statistically significant taxa diversity in microbiome composition was observed within the UC and CD groups.

**Conclusions:**

In conclusion we have shown that there are significant differences in gut microbiota between UC, CD and controls in a Saudi Arabian inflammatory bowel disease cohort. This reinforces the need for further studies in large populations that are ethnically and geographically diverse. In addition, our results show the potential to develop classifiers that may have add additional richness of context to clinical diagnosis of UC and CD with larger inflammatory bowel disease cohorts.

**Supplementary Information:**

The online version contains supplementary material available at 10.1186/s12876-023-02904-2.

## Background

A complex and dynamic microbial community within the gastrointestinal tract regulates host metabolic and immune functions [1]. Collectively these gut microbiotas provide a wide range of physiological and immunological functions that can have significant contributions to sickness or health across a variety of conditions [2]. Crohn’s disease (CD) and ulcerative colitis (UC) are chronic immune-mediated disorders of the gastrointestinal tract falling under an overarching category of inflammatory bowel diseases (IBDs). It is evident that the pathological processes of IBDs represent a complex interplay of genetic and environmental factors and can skew immune responses against normal intestinal flora [3, 4]. The resulting intestinal dysbiosis, where loss of beneficial bacteria and diversity is evident, along with expansion of potentially pathogenic bacteria, has been widely characterized in IBD and is postulated to influence the onset and perpetuation of inflammation within the gut [5–7]. Several factors contribute to intestinal dysbiosis, including diet and lifestyle factors, host genetics and medications [8–13]. Low fiber/high fat and sugar diets reduce gut microbiota diversity and also promote pathogenic species expansion [13–16]. Diet modification is often used to reduce inflammation episodes thereby reducing IBD-related symptomology [17].

Saudi Arabia has undergone significant changes in industrialization and lifestyle over the last four decades which has greatly impacted dietary and sedentary behaviors [18, 19]. Prior to the 1980’s, IBD was considered to be rare in Saudi Arabia and surrounding regions but the incidence of IBD has been gradually increasing over the last four decades [20]. Prospective studies in Saudi Arabian populations observed an incidence of 5 per million and prevalence of 50 per million for IBD in children from 1993 to 2002 [21]. The annual incidence of the CD was observed to be 3.2 cases per million from 1983 to 1992 and rising to 16.6 cases per million /from 1993 to 2002 [22]. Dietary factors, medication and smoking are postulated to contribute to the increased CD pathogenesis in Saudi Arabia [23]. A retrospective hospital-based study of 312 Saudi IBD subjects from 1970 to 2008 showed the mean age of patients with IBD was 25.5 (SD 10.6) years. Recently, a report by Al-Amrah et al. (2023) reported the composition of gut microbiota in patients with IBD [24]. However, these results were not sub-classified into UD and CD and the study sample was small (11 patients of UC and CD). Even though some of the results are similar, a comparison cannot be made due to the reasons above. Given the rapidly rising incidence of UC and CD in this population, and also considering the scarcity of microbiome studies in Saudi populations, we performed a large-scale microbiome profiling study of Saudi patients with UC and CD in order to characterize dysregulated microbiota in this population and compare to other populations.

## Methods

### Study populations

Between 2015 and 2019, stool samples and data were collected from 219 IBD subjects (CD or UC) attending the Internal Medicine Clinics, King Fahd Hospital of the University, Al-Khobar and King Fahad Hospital, Alhafof, Saudi Arabia. Diagnosis of IBD was based on endoscopy (for CD) or colonoscopy (for UC) together with imaging studies [25]. The inclusion criteria included patients over the age of 18 years who had a clinical diagnosis of IBD through endoscopy or colonoscopy examinations. Patients were excluded from the study if they had intestinal cancer, *H. pylori* infection, or had been prescribed antibiotic treatment in the two-month period prior to the date of inclusion in the study. Equivalent samples and data were also derived from 124 healthy controls. The control population did not have any evidence of T2D from HbA1C readings or from physician notes, nor did they have a family history of T2D. Participants who had been treated with antibiotics in the previous three months, were pregnant or lactating, or had a metabolic disease were excluded from the study.

Ethical approval of the study was obtained from the Abdulrahman Bin Faisal University Institutional Review Board (IRB-2019-01-115) and the study was conducted according to the ethical principles of the Declaration of Helsinki and Good Clinical Practice guidelines. All participants provided signed written informed consent.

### DNA extraction and preparation

Stool samples were taken from IBD (n = 219) and healthy (n = 124) participants. Bacterial DNA extraction from stool samples was performed using QIAamp Fast DNA Stool Mini Kit (Qiagen, Hilden, Germany) according to the manufacturer’s instructions.

### Methods for DNA Library generation and sequencing

Libraries were prepared using the Swift Amplicon 16 S rRNA Panel according to the manufacturer’s instructions and including SNAP Combinatorial Dual Indexes for multiplexing (Integrated DNA Technologies [IDT], Coralville, IA, USA). Bead-based library normalization and pooling was performed using Swift Normalase (IDT, Coralville, IA, USA), and representative sets of libraries were assessed for quantity and quality.

### 16 S microbiome sequencing

We utilized a sequencing strategy that targets all variable regions in the 16 S rRNA gene. This was carried out using the Swift Amplicon 16 S rRNA Panel (IDT, Coralville, IA, USA) to enable strain-specific identification of microbial species. The assay utilizes a pool of five overlapping primer pairs for a total targeted area spanning V1-V9, and the resultant libraries are suitable for sequencing on Illumina NovaSeq 6000. The indexed libraries were on average 620 base pairs (bp) in length, and individual DNA libraries were diluted to 2.5 nM, pooled in equimolar proportion, and sequenced on a flow cell (Illumina, CA) using 250 bp paired-end reads. Taq PCR Master Mix from Qiagen was used to prepare the PCR master mix. The three PCR products from each sample were pooled together.

### Analyses

Illumina software was used for deconvolution the initial primer and barcode processing of all raw sequences. Raw sequences were demultiplexed with Illumina’s bcl2fastq2 v2.20 Seqtk [26]. FastQC was then used for further processing to remove samples with low quality scores across the majority of bases [27]. After de-multiplexing the raw sequences and screening via FastQC, the majority of data processing was executed in Quantitative Insights into Microbial Ecology Version (QIIME2) with custom scripts [28]. Paired-end reads were joined using the *VSEARCH* function [29]. Chimera amplicon removal and abundance filtering were processed using *Deblur* [30]. Amplicon sequences were clustered and assembled into OTUs using closed reference clustering against the Greengenes (13_8 database) using *VESEARCH*[]. Taxonomic assignment was performed using a pre-trained Naïve Bayes classifier with Greengenes OTU database. The abundance tables and data obtained from *QIIME2* were combined into a *Phyloseq* object (version 4.1.1), normalized for library size variation using *DADA2* (ttps://github.com/benjjneb/dada2), and further analyzed in R with custom scripts [31]. Within each sample, we calculated the relative abundance of each phylum for each UC, CD and control group and biological sex. Next, we evaluated alpha- and beta-diversity for all groups and calculated the Shannon diversity index.

We calculated the number of meaningful principal components to retain using the broken-stick test implemented in the package *PC-Dimension* (v 1.1.11). Briefly, the broken-stick test simulates a series of principal components corresponding to random variation and only the empirical principal components explaining more variation than those generated by the broken-stick model are retained. We visualized samples in PCA space using all combinations of meaningful principal components. Second, we calculated the Bray-Curtis dissimilarity, a measure of the dissimilarity in taxonomic composition between groups, using the vegan package. We visualized the Bray-Curtis dissimilarity between all pairwise combinations and annotated samples by UC, CD and control group status and sex using the *pheatmap* package (v 1.0.12). All other visualizations were created with the *ggplot2* package and custom scripts (version 3.3.5) to cull poorly sequenced reads (https://ggplot2.tidyverse.org.).

Principal coordinates analysis (PCoA) was performed to evaluate the differences in microbial community structure across sample types. Briefly, the *phyloseq* package (1.38.0) was used to calculate the relative abundance of each OTU, from that a Bray-Curtis dissimilarity matrix was generated using the vegan package (v 2.5-7). A PERMANOVA model was used to assess the effect diagnosis, sex, diabetes status, region of sample collection (area), family history of IBD, number of family members with IBD, and nationality had on the beta diversity utilizing all patients grouped together. Patients were then assigned to one of three individual bins based on age (0–27 years, N = 75 patients, 28–36 years, N = 74 patients, and > 36 years, N = 70 patients. The same analysis was then repeated on the patients from the respective age bins assessing the effect that the aforementioned variables had on beta diversity.

### Differential abundance testing

The *DESeq2* package (v 1.32.0) was used to test for differential abundance of OTUs by modeling counts using the negative binomial distribution. Custom R scripts were used to process the abundance and metadata to create a DESeq2 object and design formula that modeled the *DESeq2* normalized counts as a function of the area, nationality, diagnosis, diabetes status, and age bin.

### Data Quality Control and Filtering

For the 16 S sequencing data from IBD and healthy controls, most samples generated sufficient reads for downstream analyses, with mean number of reads per sample and median number of reads per sample being 1,198,986 and 1,182,939, respectively (Additional File 1: Figure. S1). Principal component analysis (PCA) was performed, and the results of the broken-stick test indicated that 2 principal components were most meaningful to retain (explaining 9% and 3% of the variance, respectively). The scatterplots were annotated using various categorical variables from the metadata: area, nationality, family history, number of family members affected, sex, diagnosis, and diabetes status (Additional File 2: Fig. S2). Sample pruning using read filters showed that the middle 50% of the distribution of reads per sample was sufficient (917,332–1,474,985), although a small number of samples were observed with relatively low read counts that could bias downstream results (Additional File 1: Fig. S1). To determine the appropriate read count filter to prune samples, a tabulation for the number of samples to be retained was generated using filters ranging from 0 to 150,000 reads in increments of 5,000 and plotted the results (Additional File 3: Fig. S3). Based on the results of the read filter comparison, only samples with at least 25,000 reads were retained for downstream analyses, bringing the total number of samples in the data set to 227.

An evaluation of the number of Operational Taxonomic Units (OTUs) was performed to facilitate removing and reducing the burden of multiple testing correction in downstream analyses using a prevalence filter. Within each phylum, we plotted the prevalence of each OTU as a percentage against the total abundance of all OTUs in the data set (Additional File 4: Figure. S4). To determine the most appropriate prevalence filter which balanced removing rare taxa while preserving those relevant to the biological questions of interest, the minimum number of samples in which an OTU had to be observed was computed using a range of prevalence filters. Specifically, we tested prevalence filters from 0 to 100% in increments of 5% and plotted the number of OTUs retained in a barplot (Additional File 5: Figure. S5). A dramatic decrease in the number of OTUs retained was observed after applying a 5% prevalence threshold. To maximize statistical power in downstream analyses, a 10% prevalence filter was chosen, leaving a total of 2,502 OTUs in the taxa-pruned data set.

Next, we evaluated the proportion of uncharacterized OTUs at each taxonomic level (Additional File 6: Table S1). One OTU was uncharacterized at the phylum level and was removed from the data set. Reads were agglomerated at the genus level, as approximately 41% of OTUs were uncharacterized at the species level, resulting in a data set with 198 distinct OTUs.

### Unsupervised discovery methods

To determine whether there were distinct differences between the different subpopulations that would drive overall classification using unsupervised methods, PCA was performed using the *phyloseq* package and on a Bray-Curtis dissimilarity matrix using the *vegan* package. The scatterplots were annotated using various categorical variables from the metadata: area, nationality, family history, number of family members affected, sex, diagnosis, and diabetes status (Additional File 7: Figure. S6.1-S6.21). Scatterplots of individual coordinates on principal coordinates axes 1 and 2 which explained 24% and 14% of the variance, respectively.

### Supervised Discovery

We performed specific pairwise differential abundance comparisons to ask whether specific OTUs were significantly associated with CD, UC and control participants. The *DESeq2* package (v 1.32.0) was used to test for differential abundance of OTUs. Custom R scripts were used to process the abundance and metadata to create a *DESeq2* object and design formula that modeled the *DESeq2* normalized counts as a function of the area, nationality, diagnosis, diabetes status, and age bin.

## Results

There were 135 and 84 individuals with CD and UC, respectively, with microbiota datasets available for analyses (Table 1). In the overall 219 IBD cases, 8 (3.6%) and 6 (2.7%) of subjects had confirmed or borderline Type-2 Diabetes (T2D), respectively, as assessed by HbA1C readings and physician notes. The mean age in the IBD cases and controls was 34.0 (± SD = 11.7) and 46.8 (± SD = 9.9). Family history of T2D was evident in 16 IBD subjects (7.3%) (Table 1).

**Table 1:** Clinical and demographic characteristics for Saudi Arabian IBD cases (244) and controls (124)

	All	IBD cases	Controls	p-value		
N	343	219	124			
Age (Mean ± SD)	57.5 ± 12.5	34.0 ± 11.7	46.8 ± 9.9	< 2.2e-16		
Sex N (%) Male	219 (63.8%)	143 (65.2%)	76 (61.2%)	0.86		
Type-2 Diabetes N (%)						
Yes N (%)	8 (2.3%)	8 (3.6%)	0 (0%)	0.013		
Borderline N (%)	6 (1.7%)	6 (2.7%)	0 (0%)			
Family history N (%)	16 (4.6%)	16 (7.3%)	NA	--		
Diagnosis						
Crohn’s N (%)	135 (39.3%)	135 (61.6%)	NA	--		
Ulcerative N (%)	84 (24.4%)	84 (38.3%)	NA			
Normal N (%)	124 (36.1%)	NA	124 (100.0%)			
HbA1c (%) Median [IQR]	5.7 [5.1,6.1]	5.7 [5.1,6.1]	NA	--		
CRP Median [IQR]	1.2 [0.3,3]	1.2 [0.3,3]	NA	--		

Note: Statistically significant difference in CRP levels between Crohn’s Disease and Ulcerative Colitis Median [IQR]: 1.85 [0.5,3.85] vs. 0.6 [0.2,2] (p = 0.0002)

### Unsupervised discovery methods

**Fig. 1:**
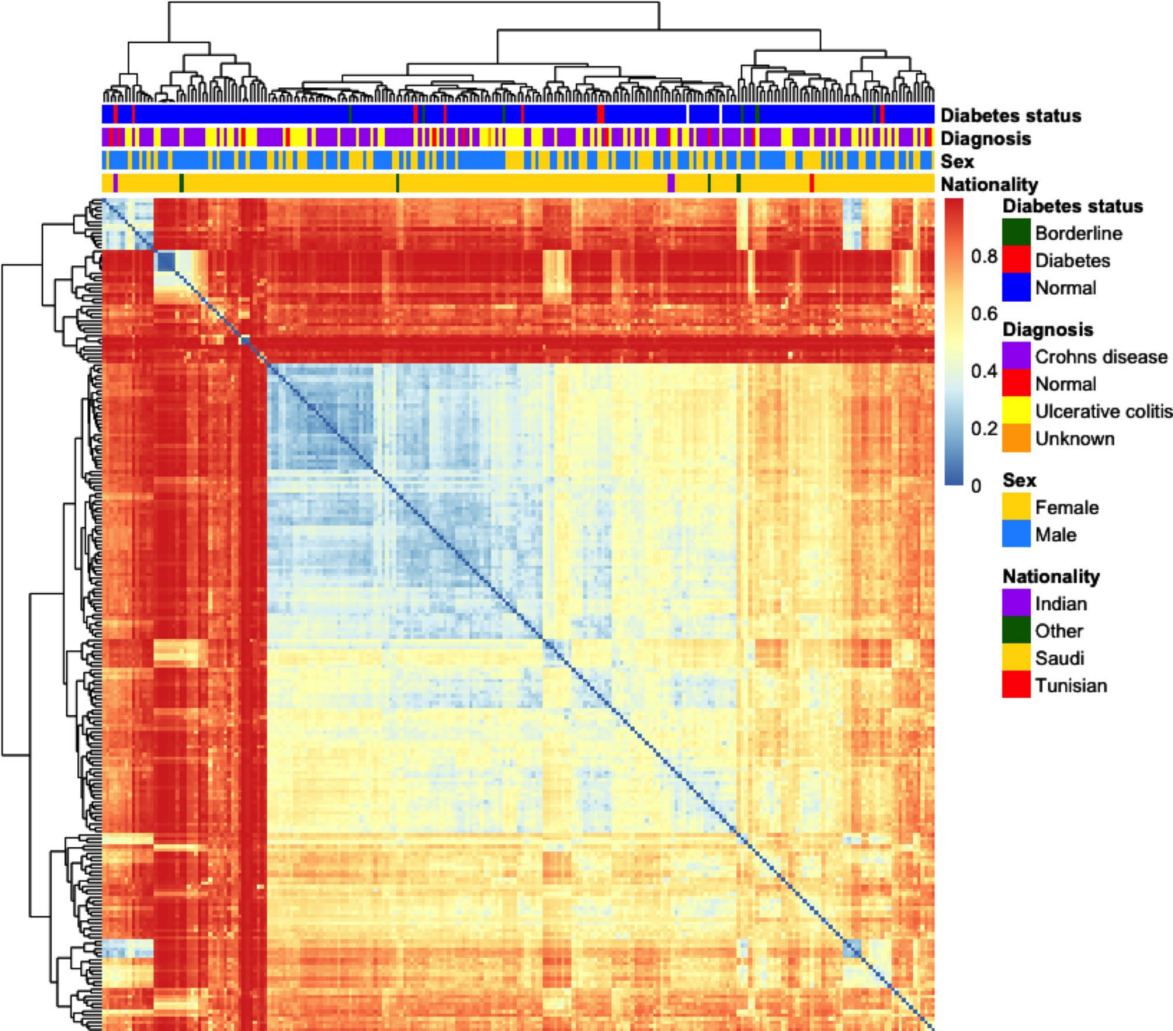
Heatmap of Bray-Curtis dissimilarity. Each sample is represented as a cell in the heatmap matrix. Color scale describes the Bray-Curtis dissimilarity, with 0 meaning the two microbial communities are the same, and 1 meaning they are completely different. Horizontal bar plots above the dendrogram are annotated with categorical fields from the metadata

We created a heatmap of the Bray-Curtis dissimilarity matrix using the *pheatmap* package and annotation of the plot was performed using the same categorical fields in the metadata except for number of family members affected (Fig. 1).

In the PCoA scatterplots, evidence of structure in the data related to differences in community composition was observed but no clear patterns associated with the disease status were evident. Evidence of structure in the heatmap was also observed, with groups of samples with clear differences in community compositions evident by the relatively high Bray-Curtis dissimilarity estimates. However, similar to the PCoA scatterplots, there was no obvious pattern evident with case diagnosis. From this we conclude that IBD status was not a main driver of microbiome dissimilarity in this population.

**Fig. 2:**
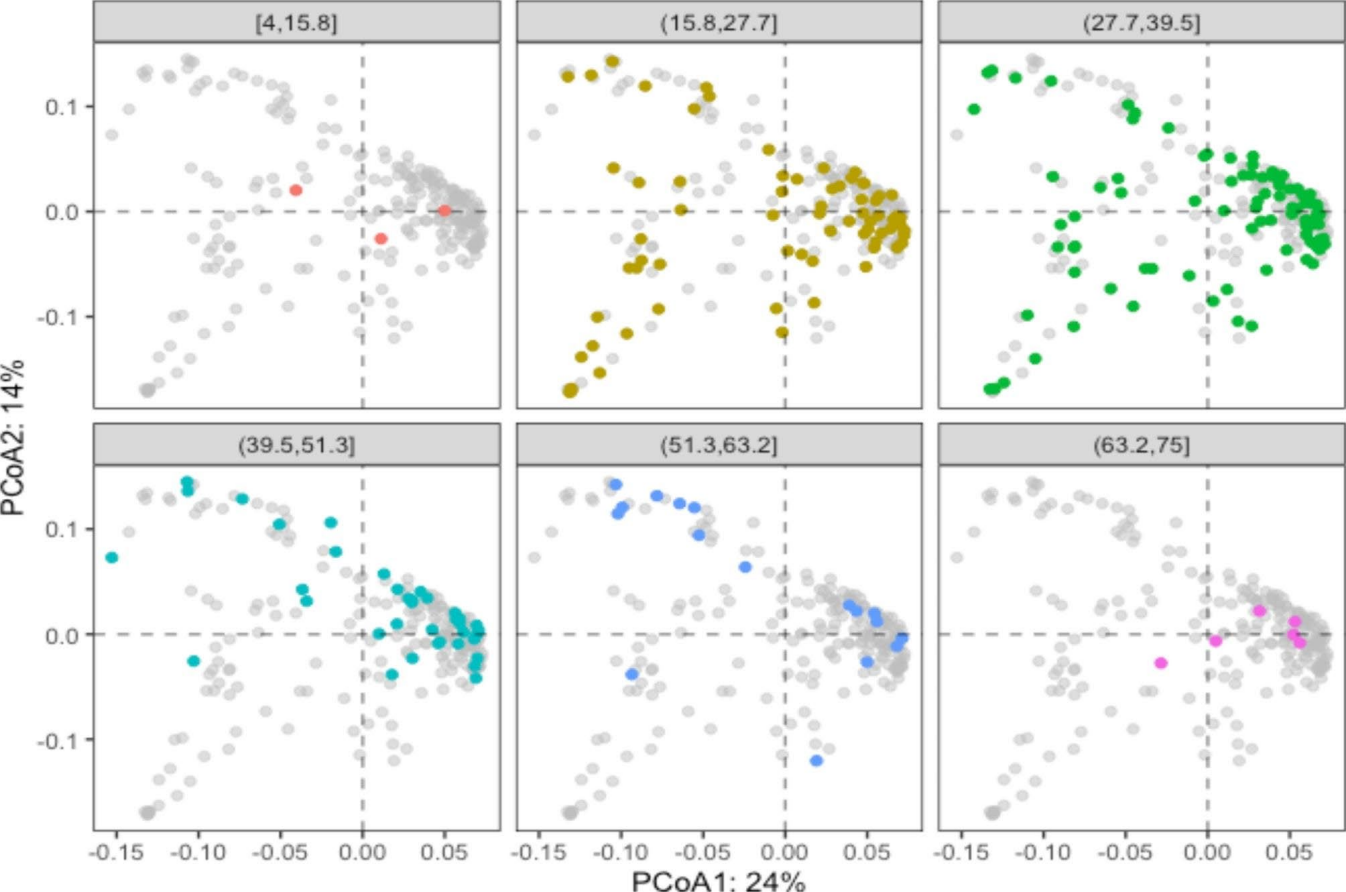
Bray-Curtis PCoA scatterplots of dissimilarity on principal coordinates axes 1 and 2. Each dot represents a sample, colored by 6 age bins with remaining samples shown in grey. Percent of variance explained by each principal coordinate is displayed on associated axis

To determine whether other population characteristics were associated with microbiome dissimilarity, various fields from the metadata were fitted onto the ordination scores from Additional File 7: Fig. S6.1-S6.21 and tested for statistical significance using a permutation test with 10,000 iterations. In addition to the categorical fields described in previous figures, age was also included. Age was significant *(p*-value = 5e-04), but only explained approximately 7% of the variation in the data (R^2^ = 0.0665). In addition, sex was significant (*p*-value = 0.0379), but only explained approximately 1.5% of the variation in the data (R^2^ = 0.0147). None of the other categorical metadata variables, including case diagnosis, were statistically significant. Given that age was statistically significant in the permutation test, the PCoA scatterplot was annotated by age. First, the age was categorized variable into 6 distinct bins using the *ggplot2* package: [4-15.8], [15.8–27.7], [27.7–39.5], [39.5–51.3], [51.3–63.2], and [63.2–75]. The *gg-highlight* package (v 0.3.2) (https://cran.r-project.org/ web/packages/ gghighlight/index.html) was used to plot samples from each age bin on top of the rest of the data in a series of faceted scatterplots (Fig. 2). Given that age was determined to be a statistically significant driver of differences in beta diversity, we performed the same analysis but sorted each patient into one of three bins based on age (0–27 years, 28–36 years, and > 36 years). The only variables to show a statistically significant effect on beta diversity were family history of IBD and the number of family members affected by IBD in the 0–27 age bin (Additional Files 8,9,10,11: Figs S7, S8, S9, S10).

### Supervised Discovery

**Fig. 3:**
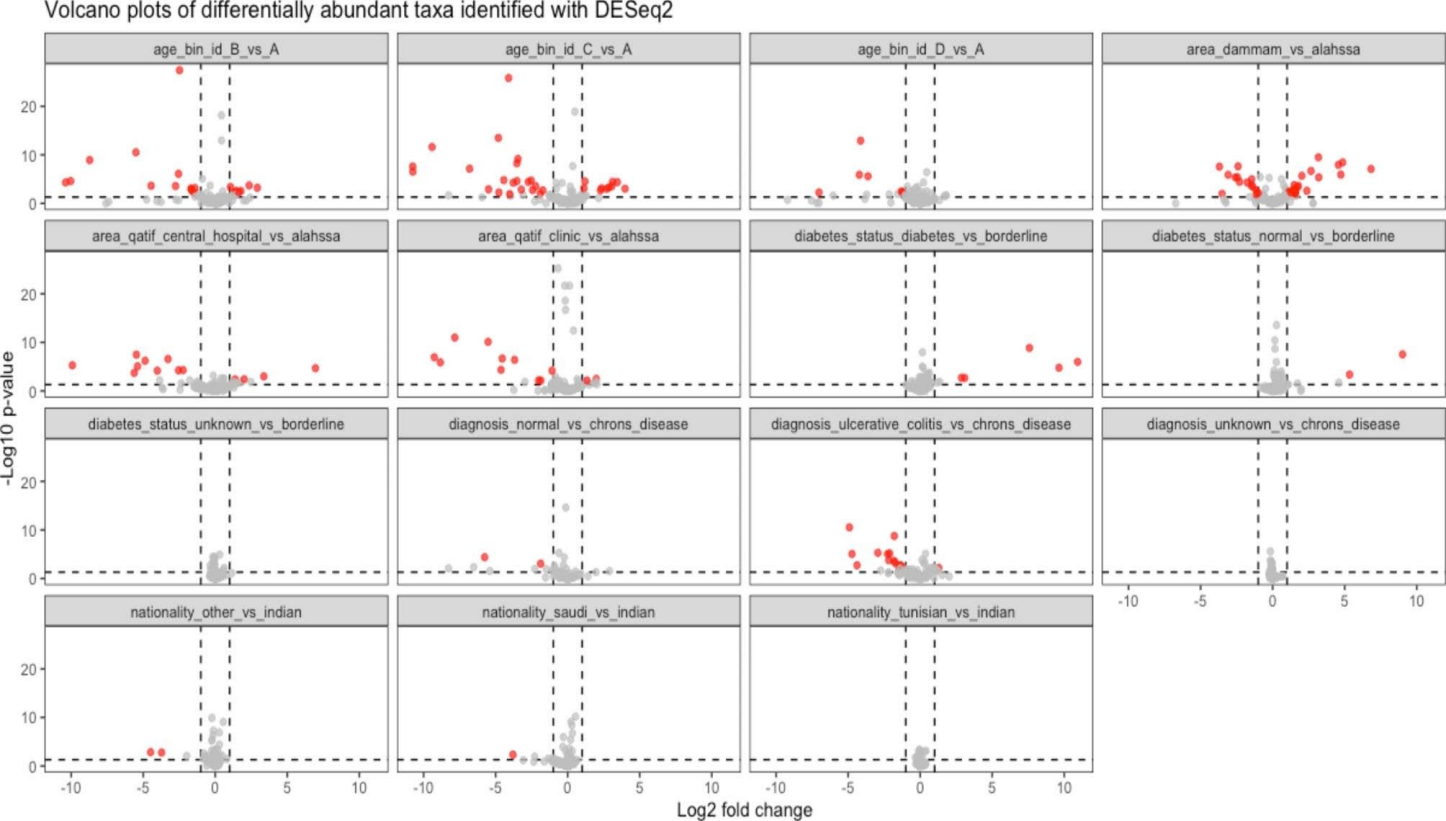
Volcano plots faceted by each comparison in *DESeq2* model with log2 fold change in abundance between groups (x-axis) and -log10 raw p-value (y-axis). Dashed vertical black lines represent − 1 and + 1 log2 fold change; the dashed horizontal black line represents the raw p-value threshold of 0.05. Each OTU is represented as a dot, with red dots representing differentially abundant OTUs with adjusted p-values < 0.05 and absolute value of log2 fold change estimates > 1

Figure 2 illustrates the *DESeq2* normalized counts as a function of the area, nationality, diagnosis, diabetes status, and age bin. Results for each covariate comparison were tabulated in the model formula and shrunk the log2 fold change estimates using the *apeglm* function in DESeq2, resulting in a total of 294 differential abundant OTUs across all comparisons (adjusted *p*-value < 0.05 using Benjamini and Hochberg FDR method). We created volcano plots faceted by each comparison in the model to visualize the effect size of differential abundances estimates and p-values (Fig. 3).

**Fig. 4:**
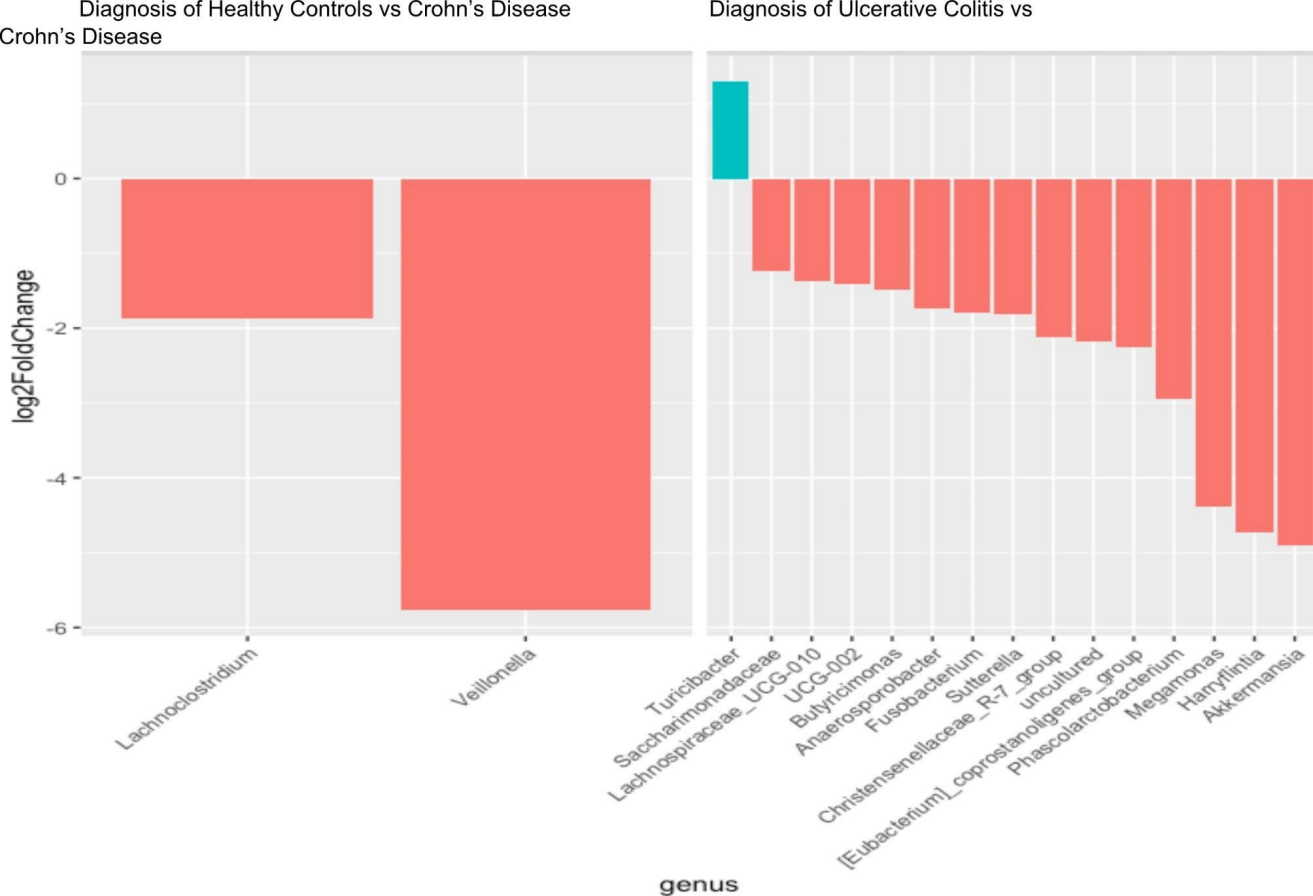
Barplot of statistically significant differentially abundant OTUs for disease diagnosis comparisons (adjusted p-value < 0.05). Genus associated with each OTU (x-axis) and the shrunken log2 fold change estimate (y-axis_. Negative log2 fold change estimates correspond to reduced abundance of each OTU in the Healthy controls (left plots) and CD samples (right plots)

Each OTU in the plot is represented as a dot, with red dots representing differentially abundant OTUs with adjusted p-values < 0.05 and absolute value of log2 fold change estimates > 1. Figure 4 illustrates comparisons across IBD disease diagnoses, with the differentially abundant OTUs shown as barplots. Negative log2 fold change estimates correspond to reduced abundance of each OTU in the normal and UC samples in the left and right plots, respectively. In contrast, positive log2 fold change estimates correspond to increased abundance of each OTU in CD samples in both plots.

**Fig. 5:**
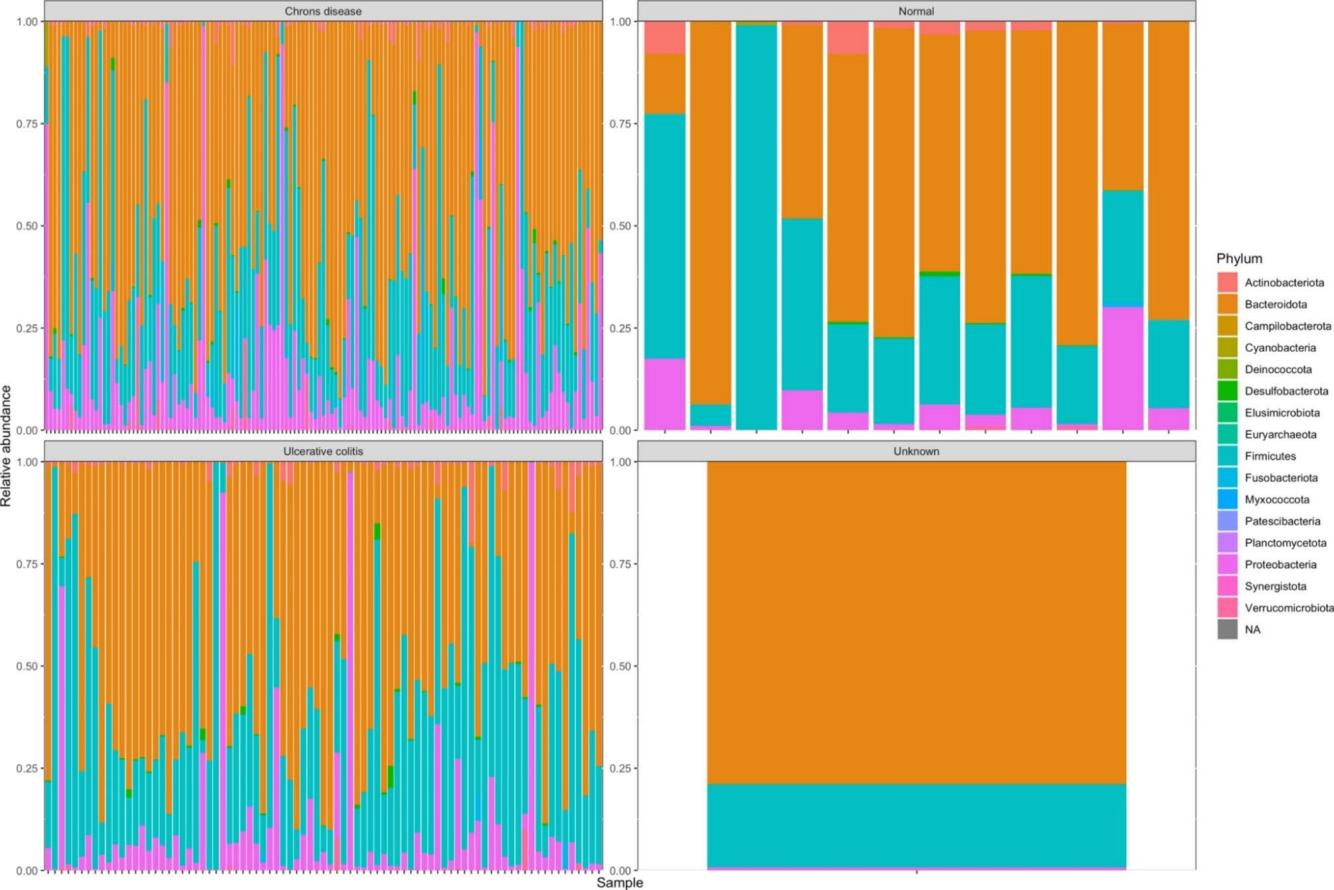
Phylum-level OTU abundance across CD, UC, Healthy Controls and unknown/unclassified samples

At the genus level, two genera in particular, *Veillonella* and *Lachnoclostridium* showed significant association with CD versus controls (Fig. 4). There were significant differences between subjects with CD versus UC, with the top differential genera spanning *Akkermansia*, *Harryflintia*, *Maegamonas* and *Phascolarctobacterium*. Additional Files 12 & 13: Figures S11 and S12 illustrate both the UC versus healthy, and Crohn’s versus healthy, for Species Level Differential Abundance Testing respectively, with the top 20 upregulated and top 20 downregulated species listed in Additional File 14: Supplementary Table S2 (UC versus normal) and Additional File 15: Supplementary Table S3 (Crohn’s versus normal). The full datasets are listed in Additional File 16: Supplementary Table S4 (UC versus normal) and Additional File 17: Supplementary Table S5 (Crohn’s versus normal). At the species level, both UC and CD groups had a larger abundance of *Blautia hansenii*, which has been previously shown to be associated with visceral fat accumulation [32], when compared to controls. Additionally, both CD and UC patients saw a lower abundance of multiple species of *Prevotella* which is consistent with what other studies have shown [33]. Furthermore, significant diversity in microbiome composition was observe within the UC and CD groups (Fig. 5). Overall, we conclude that key differences in microbiome composition are observed between UC and CD and healthy patients.

## Discussion

Unlike the relationship witnessed in rodent models, the host-microbe relationship in IBD patients is considerably more multifaceted and diverse. In both the CD and UC forms of the disease, dysbiosis is evident. However, whether it is the cause or the effect of inflammation in the intestinal tissue is uncertain. Therefore, further delineation of IBD requires a greater level of microorganism differentiation analysis as well as taking into account environmental, lifestyle and genetic factors.

Previous studies have characterized gut microbiomes in patients with UC and CD, however, these few studies have been performed on populations not from the Middle East. Given cultural and dietary dissimilarities in Middle Eastern versus Western populations, we hypothesized that key IBD-associated microbes may be distinctly different in Middle Eastern study participants. Indeed, in our study of Saudi Arabian participants we observed a number of microbial genera that were not well characterized in prior IBD studies. In particular, *Lachnoclostridium*, which was at higher abundance in CD versus normal in our dataset, was recently observed to be upregulated in response to a high protein diet and associated with colonic mucus thickness, though a clear link with UC or IBD in general has not been elucidated [34]. While *Harryflintia* has been recently identified as associated with a high-fat diet in mice, much less is known about specific associations with CD [35]. *Akkermansia* is commonly associated with protective effects in the gut for a variety of diseases, here we show that there is significant differential *Akkermansia* abundance in CD versus UC [36–38]. This may not be unique to the Saudi population and may reflect differential presentation and severity in CD versus UC. In particular, only a subset of OTUs were overlapping with UC versus CD characterizations by Jansson and colleagues, and as such their previously developed classifier and any other classification algorithm trained largely on mostly IBD subjects of European ancestry and may not perform well when applied to participants from the Middle East [39].

For the two genera that showed significant association with CD versus controls - *Veillonella* is a an anaerobic bacteria genus commonly associated with gut inflammation and has been previously observed at differential abundances in IBD versus healthy subjects across a number of studies [40–43]. *Lachnoclostridium* was also observed as significantly upregulated in our CD population which to our knowledge not been observed in any studies to date. One small study of CD versus UC subjects showed it was upregulated in UC patients but not CD subjects [43]. In 2019 a modestly sized 16 S rRNA microbiota study comparing two sub-types of the UC, as defined by traditional Chinese Medicine theory, showed a significant increase in *Lachnoclostridium* [38].

## Conclusions

In conclusion, we have shown that there are significant differences in gut microbiota between UC, CD and controls in a Saudi Arabian IBD cohort. This reinforces the need for further studies in large populations that are ethnically and geographically diverse. In addition, our results show the potential to develop classifiers that may have add additional richness of context to clinical diagnosis of UC and CD with larger IBD cohorts.

## Electronic supplementary material

Below is the link to the electronic supplementary material.

Supplementary Material 1

Supplementary Material 2

Supplementary Material 3

Supplementary Material 4

Supplementary Material 5

Supplementary Material 6

Supplementary Material 7

Supplementary Material 8

Supplementary Material 9

Supplementary Material 10

Supplementary Material 11

Supplementary Material 12

Supplementary Material 13

Supplementary Material 14

Supplementary Material 15

Supplementary Material 16

Supplementary Material 17

## Data Availability

The datasets generated during the current study are available in the European Variation Archive (EVA) repository (https://www.ebi.ac.uk/ena/browser/view/PRJEB57347), under the title “*Gut Microbiota Analyses of Inflammatory Bowel Diseases from a representative Saudi Population*” with accession number PRJEB57347.

## Abbreviations

CD: Crohn’s disease
UC: Ulcerative colitis
IBD: Inflammatory bowel disease
OTU: Operational Taxonomic Unit
PCA: Principal component analysis
PCoA: Principal coordinate analysis
