# Supplementary material for: Gut microbiota analyses of inflammatory bowel diseases from a representative Saudi population
Source: BMC Gastroenterol. 2023 Jul 28;23:258. doi: 10.1186/s12876-023-02904-2 (PMC10375692; doi:10.1186/s12876-023-02904-2)

**Additional File 1:Fig. S1. Sample pruning using read filters.** Histogram of the distribution of reads assigned to OTUs per sample. The red and blue dashed lines represent the mean and median number of reads, respectively.

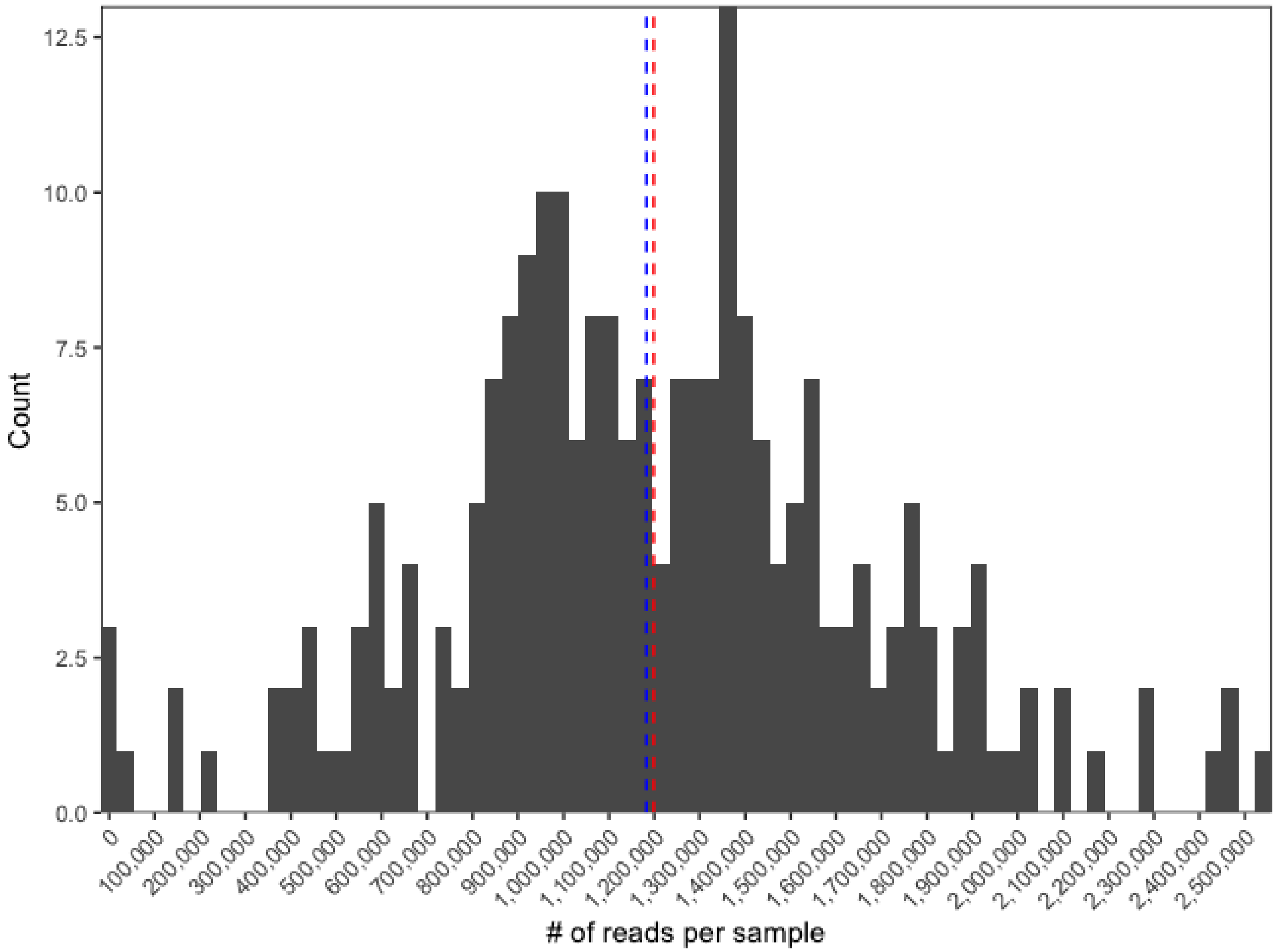

Supplement: Supplementary file 1 — Supplementary Material 1 [file 12876_2023_2904_MOESM1_ESM.pdf]
