# Supplementary material for: Gut microbiota analyses of inflammatory bowel diseases from a representative Saudi population
Source: BMC Gastroenterol. 2023 Jul 28;23:258. doi: 10.1186/s12876-023-02904-2 (PMC10375692; doi:10.1186/s12876-023-02904-2)

Additional File 2: Fig. S2. PCA scatterplots of abundance data normalized using centered log-ratio transformation on principal components axes 1 and 2. Each point represents a sample, colored by one of associated metadata fields in legend to the right of each plot. Percent of variance explained by each principal component is displayed on the associated axis.

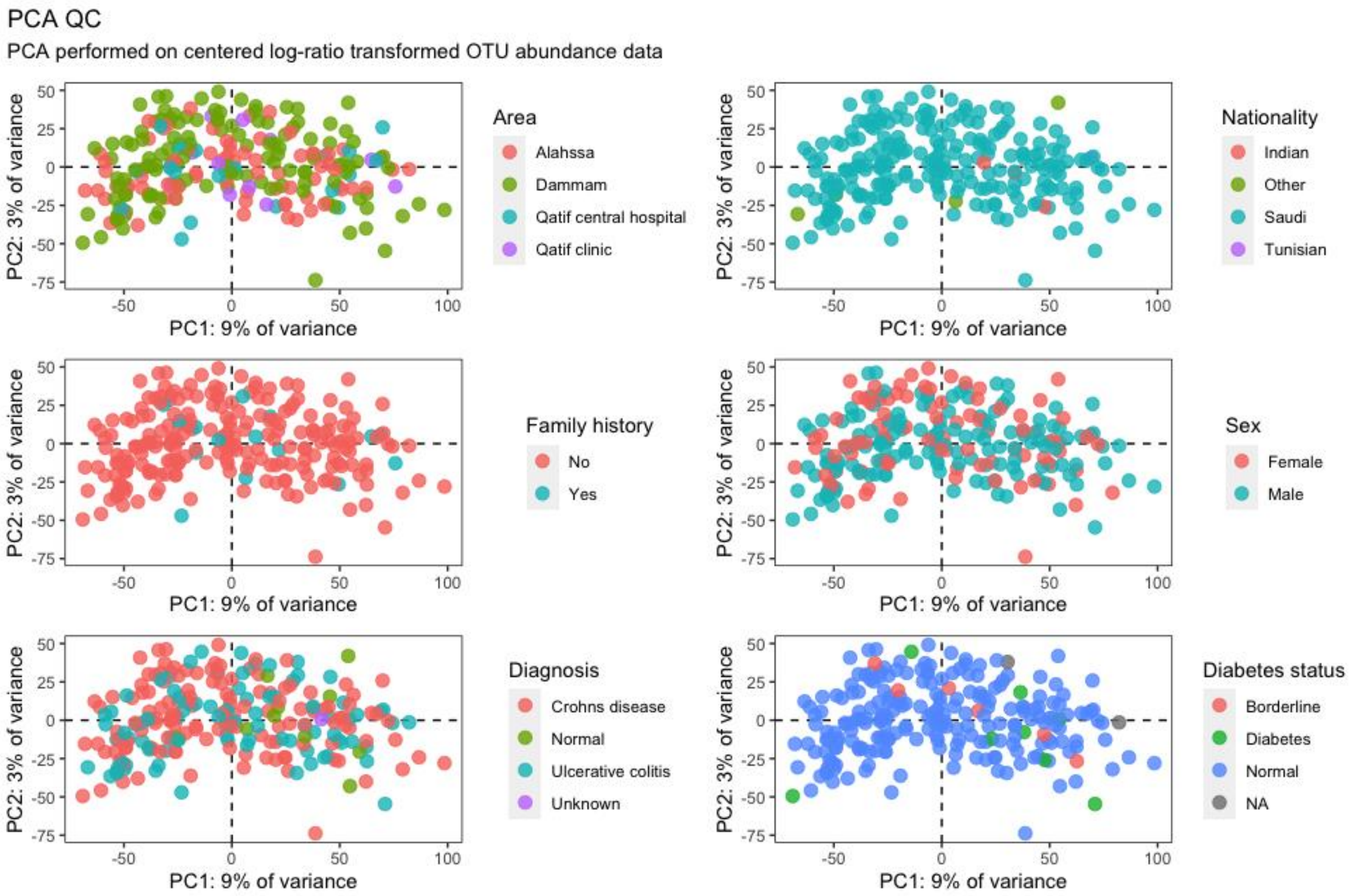

Supplement: Supplementary file 2 — Supplementary Material 2 [file 12876_2023_2904_MOESM2_ESM.pdf]
