# Supplementary material for: Gut microbiota analyses of inflammatory bowel diseases from a representative Saudi population
Source: BMC Gastroenterol. 2023 Jul 28;23:258. doi: 10.1186/s12876-023-02904-2 (PMC10375692; doi:10.1186/s12876-023-02904-2)

Additional File 3: Fig. S3. Prune samples using various filters. Line plot showing the various read filters tested (x-axis) and number of samples retained post-filtering (y-axis). Dashed vertical red line represents the point at which no additional samples are removed after increasing the read filter.

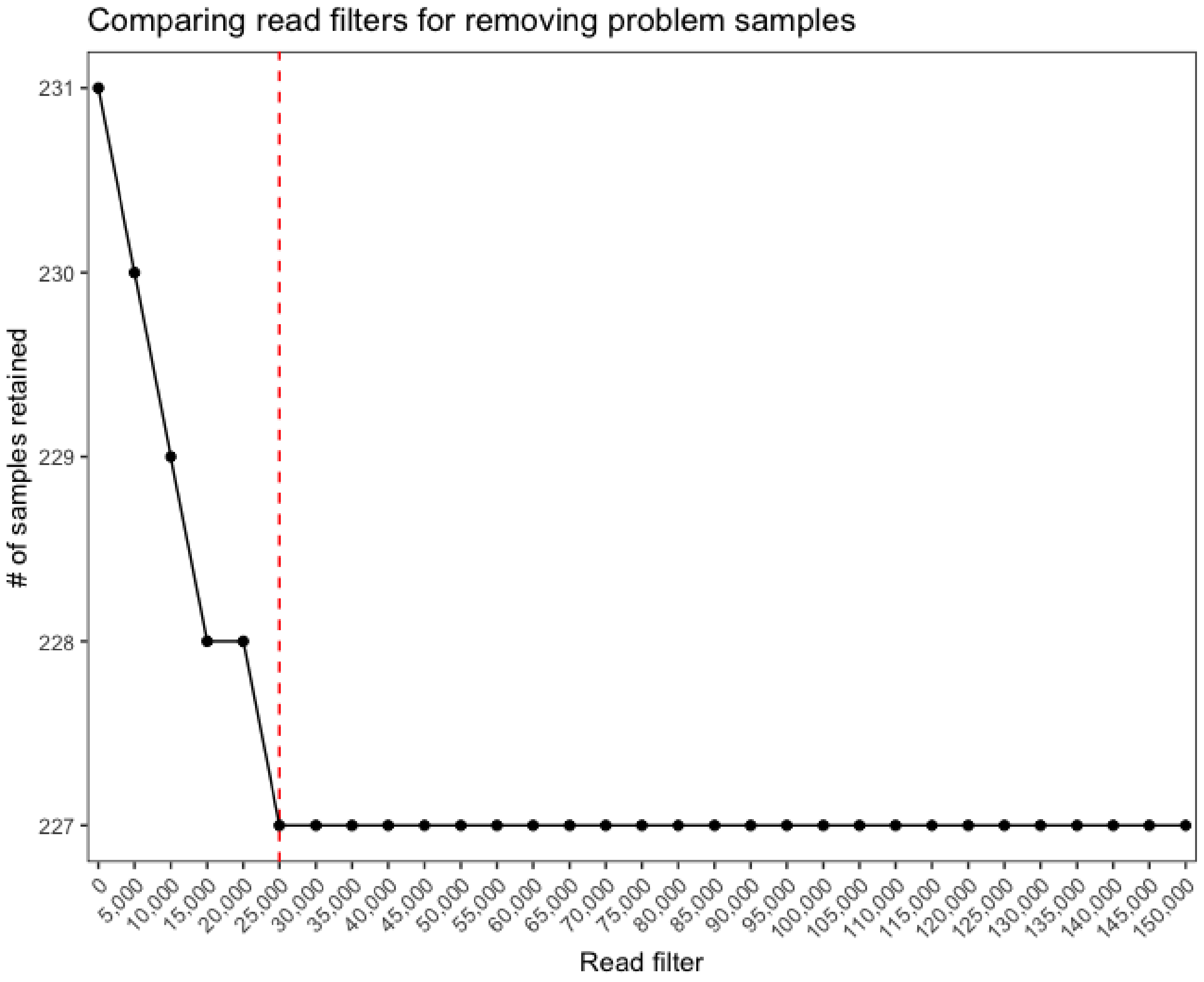

Supplement: Supplementary file 3 — Supplementary Material 3 [file 12876_2023_2904_MOESM3_ESM.pdf]
