# Supplementary material for: Gut microbiota analyses of inflammatory bowel diseases from a representative Saudi population
Source: BMC Gastroenterol. 2023 Jul 28;23:258. doi: 10.1186/s12876-023-02904-2 (PMC10375692; doi:10.1186/s12876-023-02904-2)

Additional File 4: Fig. S4. Evaluation of the number of Operational Taxonomic Units. Scatterplot showing total abundance on the log10 scale across all OTUs in the data set (x-axis) and the prevalence of each OTU as a percentage of samples where the OTU is found at least once (y-axis). Sub-plots are faceted by phylum (grey boxes). The dashed horizontal line is set at 10% prevalence.

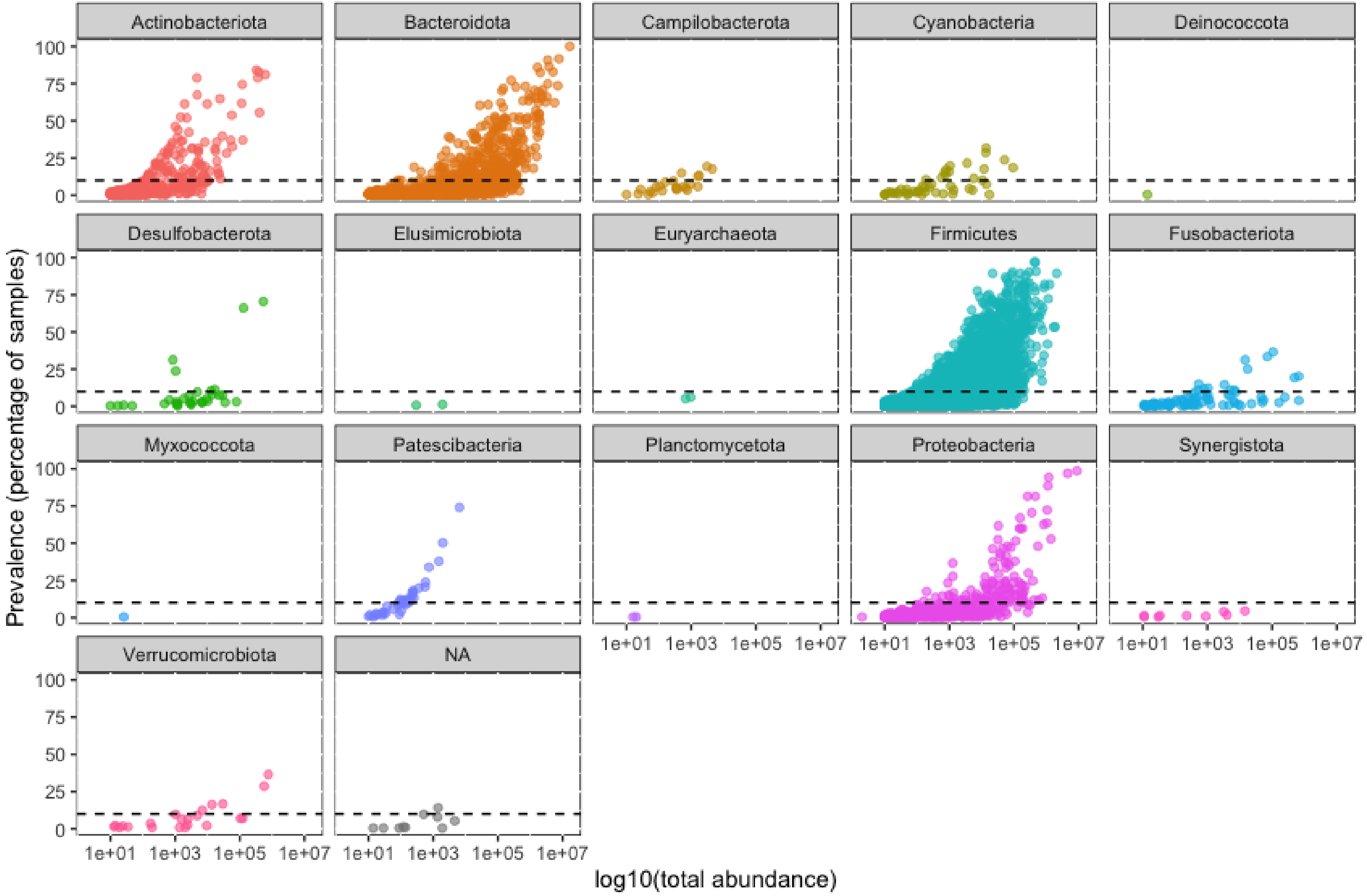

Supplement: Supplementary file 4 — Supplementary Material 4 [file 12876_2023_2904_MOESM4_ESM.pdf]
