# Supplementary material for: Gut microbiota analyses of inflammatory bowel diseases from a representative Saudi population
Source: BMC Gastroenterol. 2023 Jul 28;23:258. doi: 10.1186/s12876-023-02904-2 (PMC10375692; doi:10.1186/s12876-023-02904-2)

Additional File 5: Fig. S5. Determination of appropriate prevalence filter. Barplots showing the various prevalence filters (x-axis) and the number of OTUs retained in the data set using each filter (y-axis). Prevalence describes the percentage of samples where an OTU is observed at least once (i.e., abundance estimates > 0). A prevalence filter of 10% is represented as a vertical dashed black line.

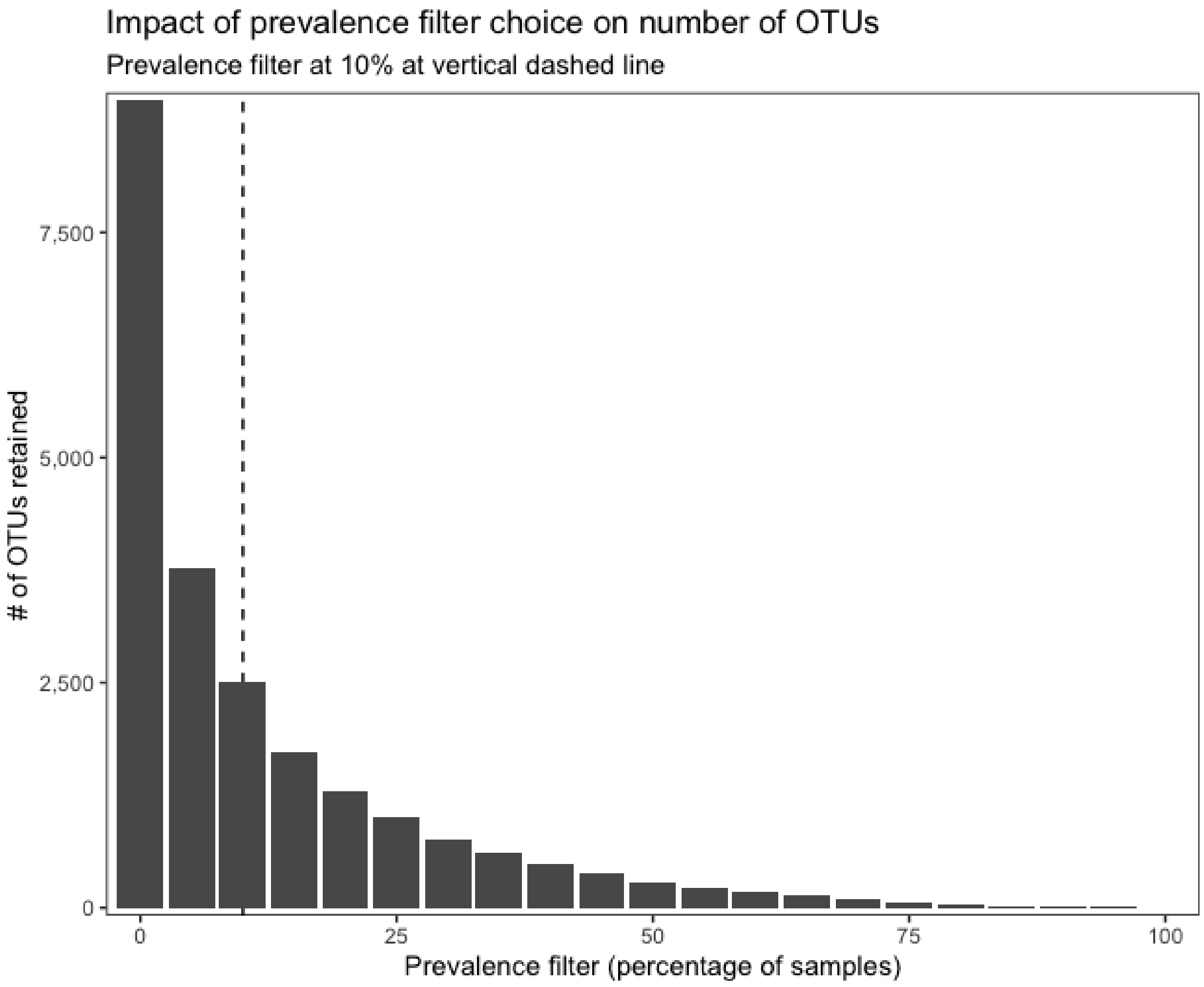

Supplement: Supplementary file 5 — Supplementary Material 5 [file 12876_2023_2904_MOESM5_ESM.pdf]
