# Supplementary material for: Gut microbiota analyses of inflammatory bowel diseases from a representative Saudi population
Source: BMC Gastroenterol. 2023 Jul 28;23:258. doi: 10.1186/s12876-023-02904-2 (PMC10375692; doi:10.1186/s12876-023-02904-2)
Supplement: Supplementary file 6 — Supplementary Material 6 [file 12876_2023_2904_MOESM6_ESM.pdf]

Additional File 6: Table S1. Evaluated of the proportion of uncharacterized OTUs at each taxonomic level. At each taxonomic level, the number of characterized and uncharacterized OTUs are presented. In addition, the proportion of OTUs that are uncharacterized is presented as well, rounded to two decimal places.

**Taxonomic level**	**# characterized OTUs**	**# uncharacterized OTUs**	**Proportion of OTUs uncharacterized**
Domain	2502	0	0.00
Phylum	2501	1	0.00
Class	2500	2	0.00
Order	2495	7	0.00
Family	2489	13	0.01
Genus	2150	352	0.14
Species	1482	1020	0.41
