# Supplementary material for: Gut microbiota analyses of inflammatory bowel diseases from a representative Saudi population
Source: BMC Gastroenterol. 2023 Jul 28;23:258. doi: 10.1186/s12876-023-02904-2 (PMC10375692; doi:10.1186/s12876-023-02904-2)
Supplement: Supplementary file 7 — Supplementary Material 7 [file 12876_2023_2904_MOESM7_ESM.pdf]

Additional File 7: Fig S6.1-S6.21. Principal coordinates analysis (PCoA) of Bray Curtis Dissimilarity for:

A. Subjects up to age 27: (1) Diagnoses (Crohn's Disease [CD], Normal or Ulcerative Colitis [UC]); (2) sex; (3) Diabetes status; (4) Region: Alahssa, Dammam, Qatif central Hospital and Qatif Clinic; (5) Family History (Y/N); (6) Number of family Affected and (7) Nationality (Saudi or Other).

B. Subjects 28-36 years of age: (8) Diagnoses CD, Normal or UC; (9) sex; (10) Diabetes status; (11) Region; (12) Family History (Y/N); (13) Number of family Affected and (14) Nationality.

C. Subjects 37 years and older: (15) Diagnoses CD, Normal or UC; (16) sex; (17) Diabetes status; (18) Region; (19) Family History (Y/N); (20) Number of family Affected and (21) Nationality. The x-axis plots PCoA 1 and the y-axis plots PCoA2 for each of these subfigures.

Fig S6.1A.

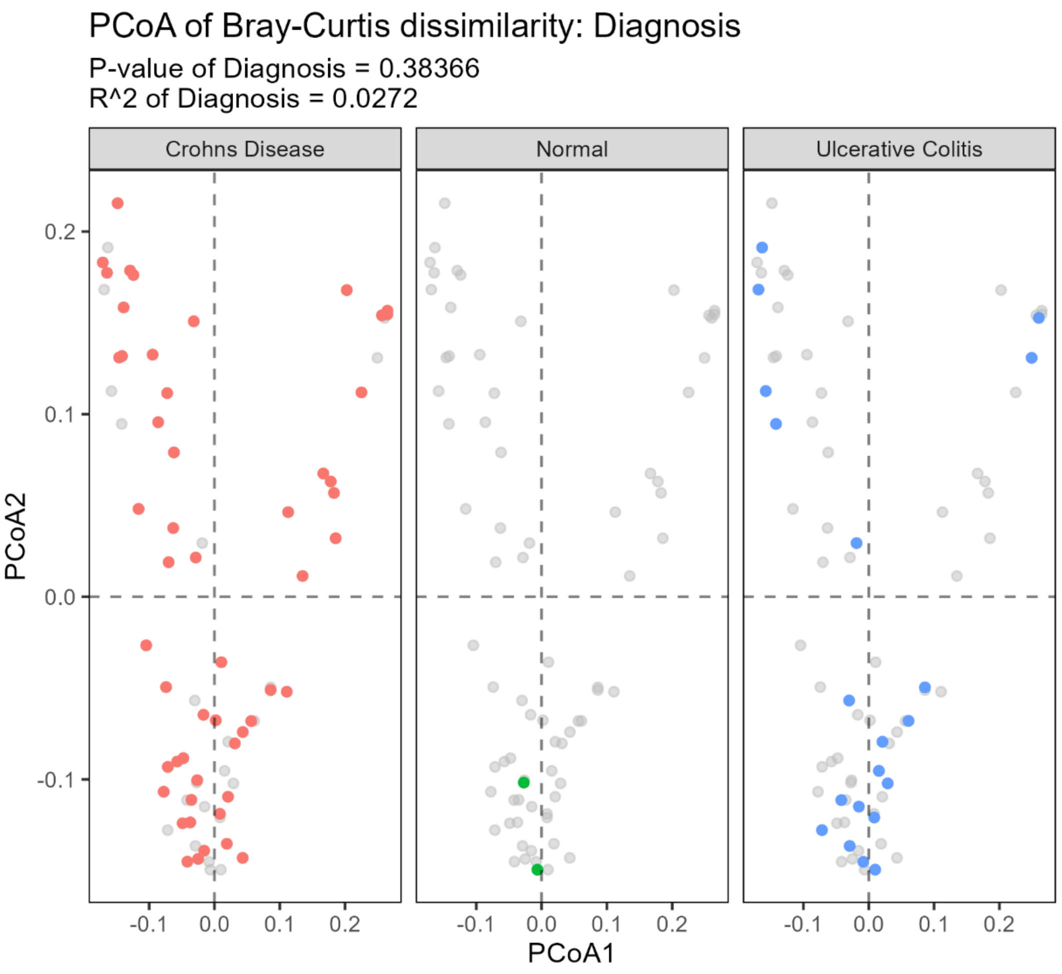
PCoA of Bray-Curtis dissimilarity: Diagnosis
P-value of Diagnosis = 0.38366
R^2 of Diagnosis = 0.0272
Crohns Disease
Normal
Ulcerative Colitis
PCoA2
0.2
0.1
0.0
-0.1
-0.1
0.0
0.1
0.2
PCoA1

Fig S6.2A.

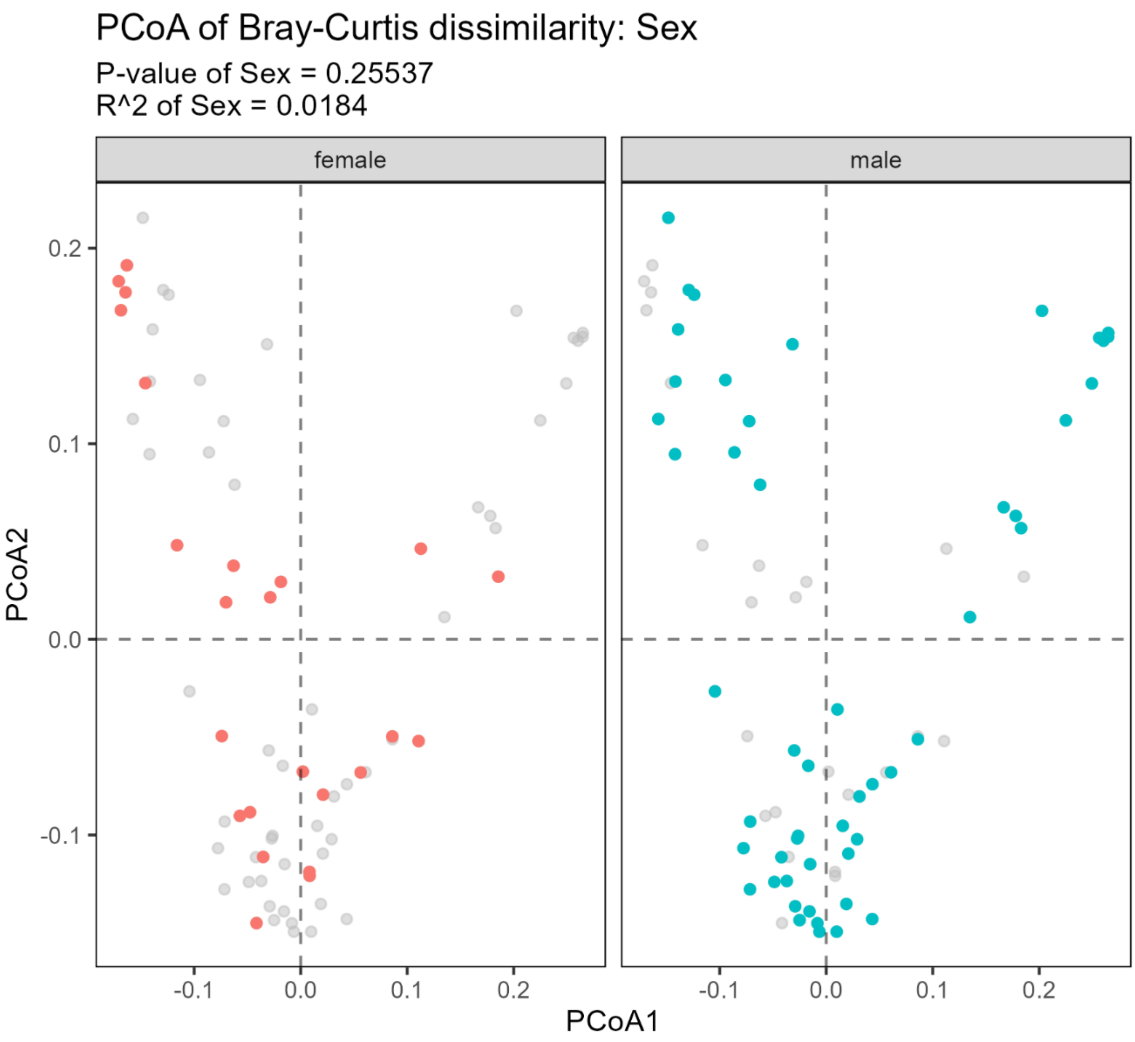
PCoA of Bray-Curtis dissimilarity: Sex
P-value of Sex = 0.25537
R^2 of Sex = 0.0184
female
male
0.2
0.1
0.0
-0.1
PCoA2
-0.1
0.0
0.1
0.2
PCoA1

Fig S6.3A

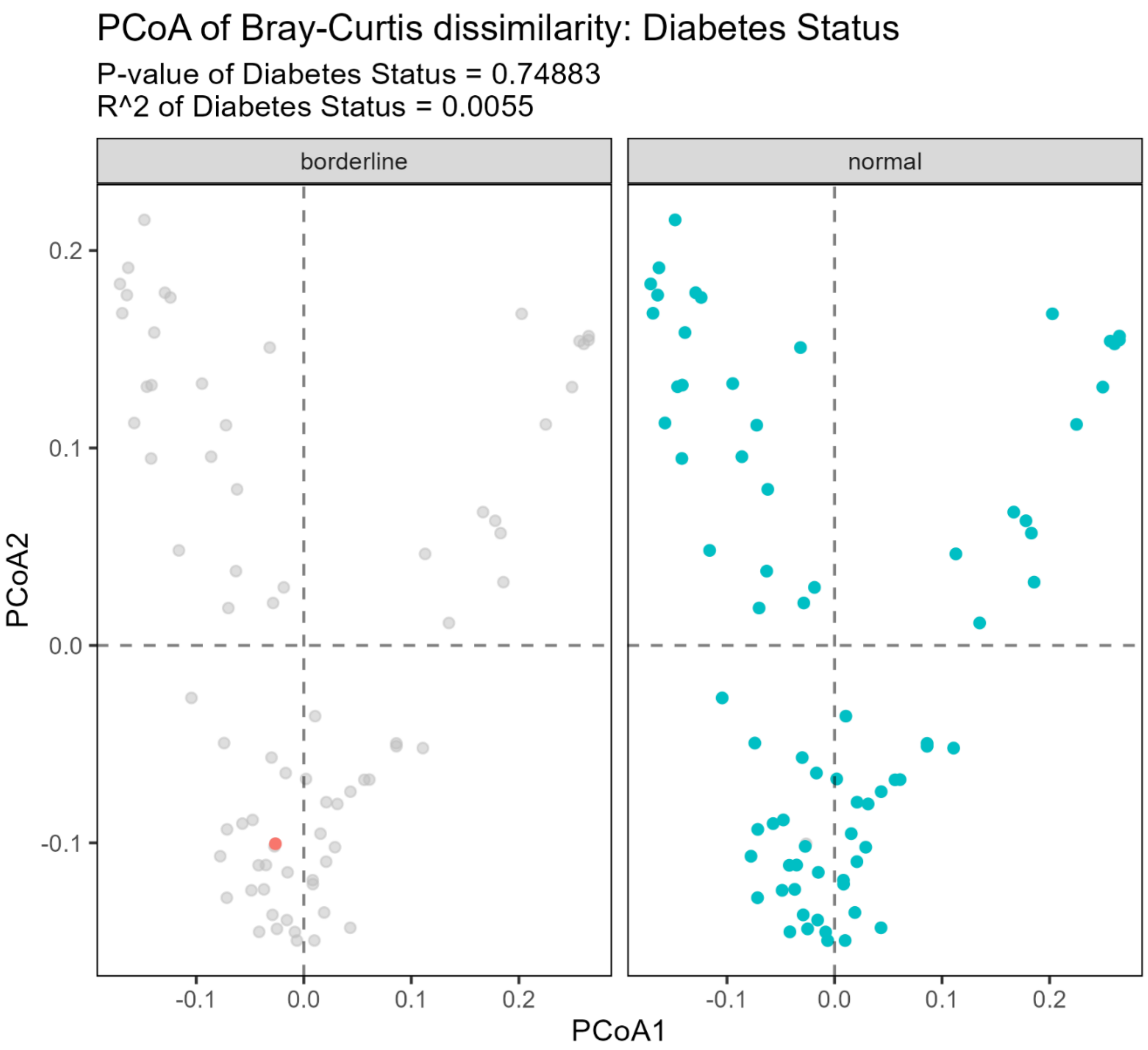

PCoA of Bray-Curtis dissimilarity: Diabetes Status
P-value of Diabetes Status = 0.74883
R^2 of Diabetes Status = 0.0055
borderline
normal
0.2
0.1
0.0
-0.1
PCoA2
-0.1
0.0
0.1
0.2
-0.1
0.0
0.1
0.2
PCoA1

Fig S6.4A

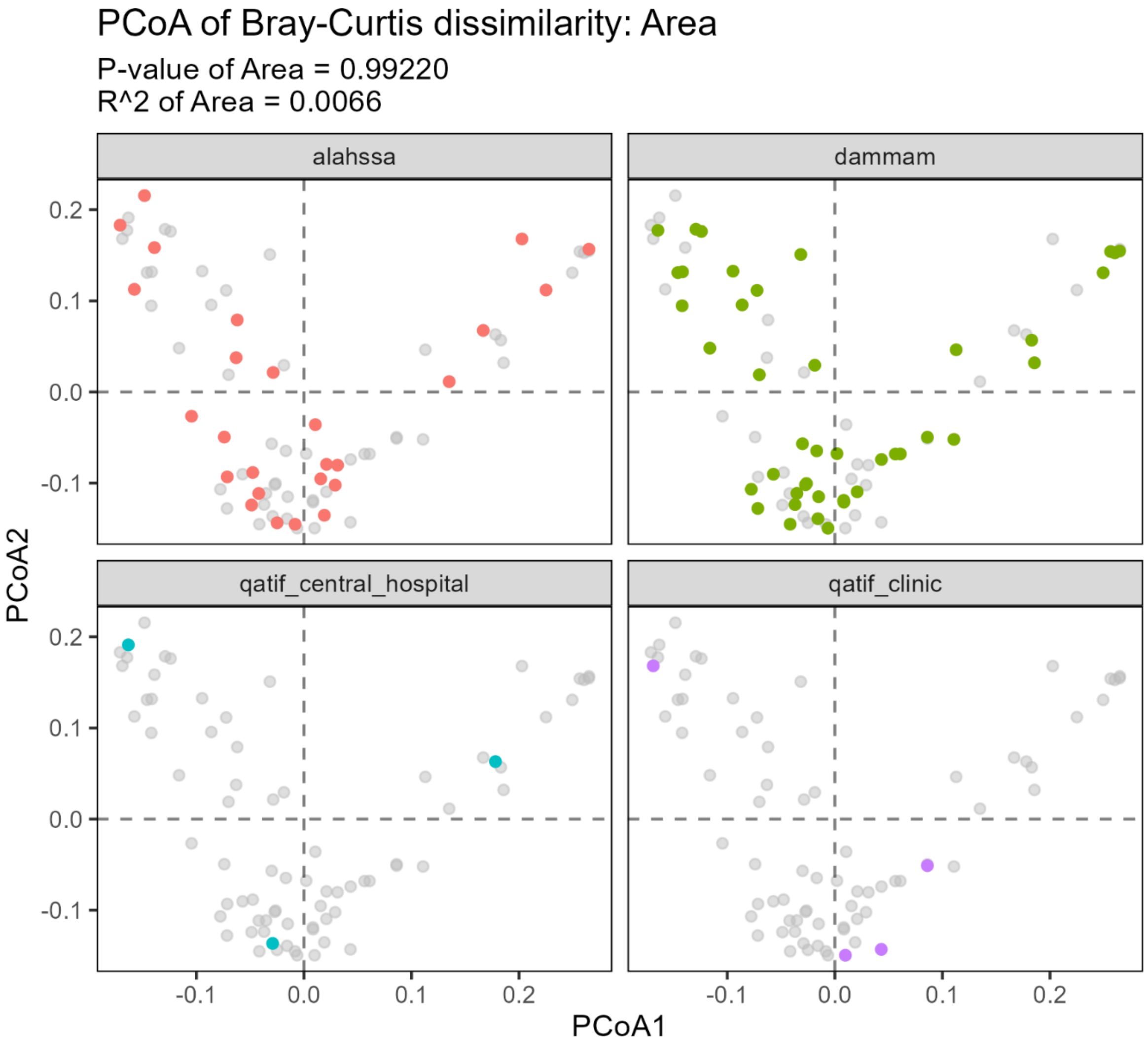
PCoA of Bray-Curtis dissimilarity: Area
P-value of Area = 0.99220
R^2 of Area = 0.0066
alahssa
dammam
qatif_central_hospital
qatif_clinic
PCoA2
PCoA1
0.2
0.1
0.0
-0.1
-0.1
0.0
0.1
0.2

Fig S6.5A

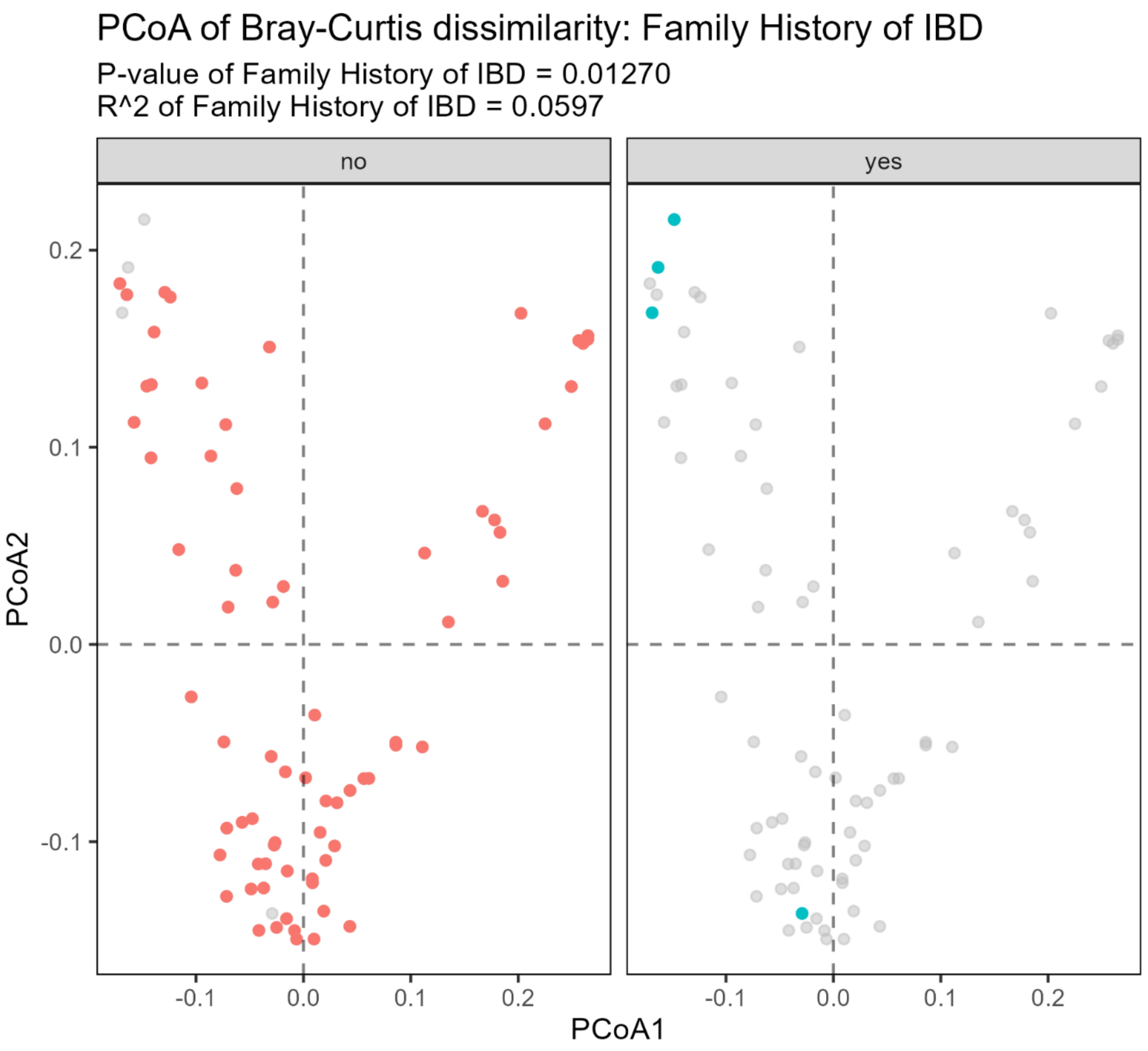
PCoA of Bray-Curtis dissimilarity: Family History of IBD
P-value of Family History of IBD = 0.01270
R^2 of Family History of IBD = 0.0597
no
yes
PCoA2
PCoA1
0.2
0.1
0.0
-0.1
-0.1
0.0
0.1
0.2

Fig S6.6A

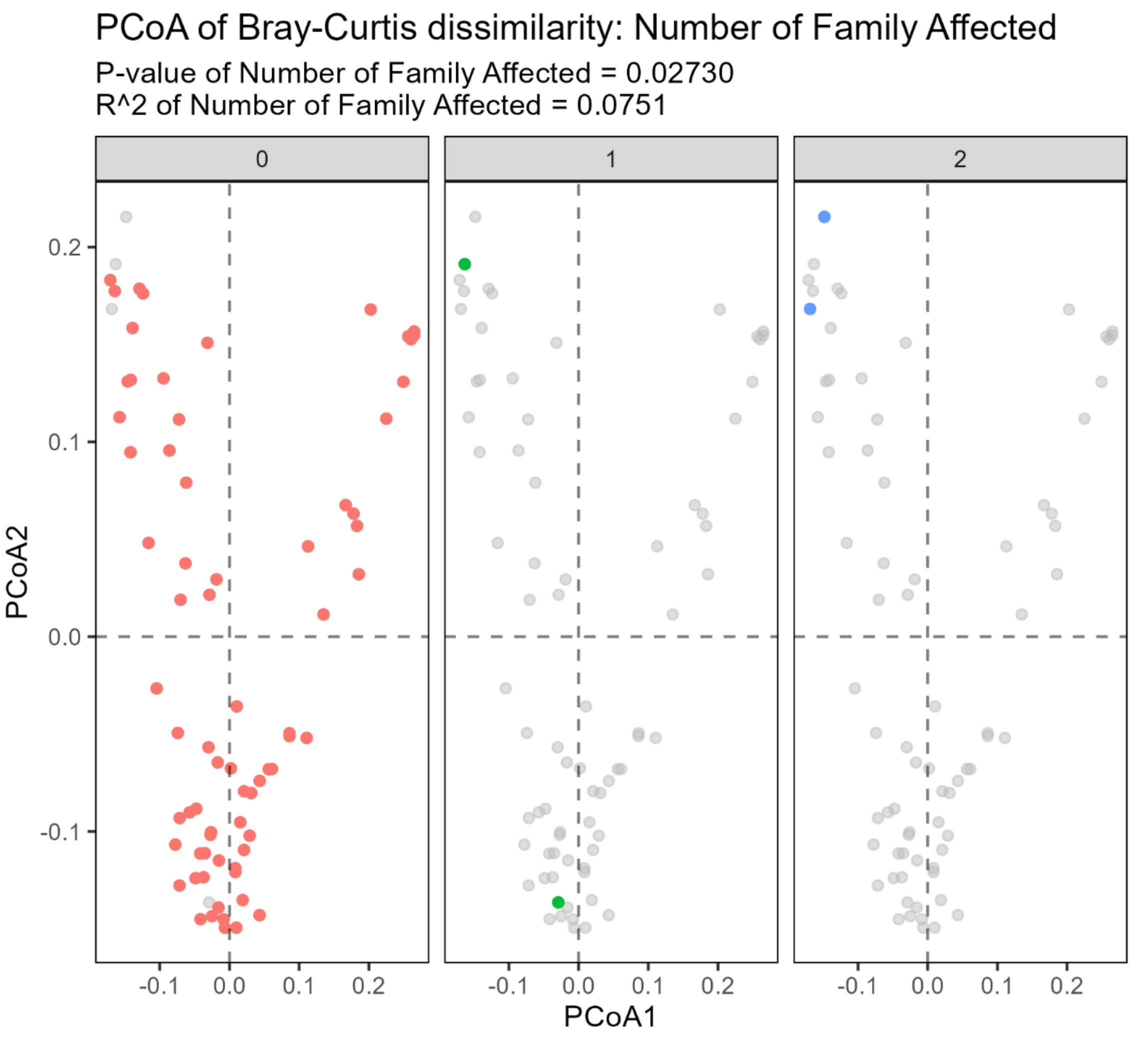

PCoA of Bray-Curtis dissimilarity: Number of Family Affected
P-value of Number of Family Affected = 0.02730
R^2 of Number of Family Affected = 0.0751
0
1
2
PCoA2
0.2
0.1
0.0
-0.1
-0.1
0.0
0.1
0.2
PCoA1

Fig 6.7A

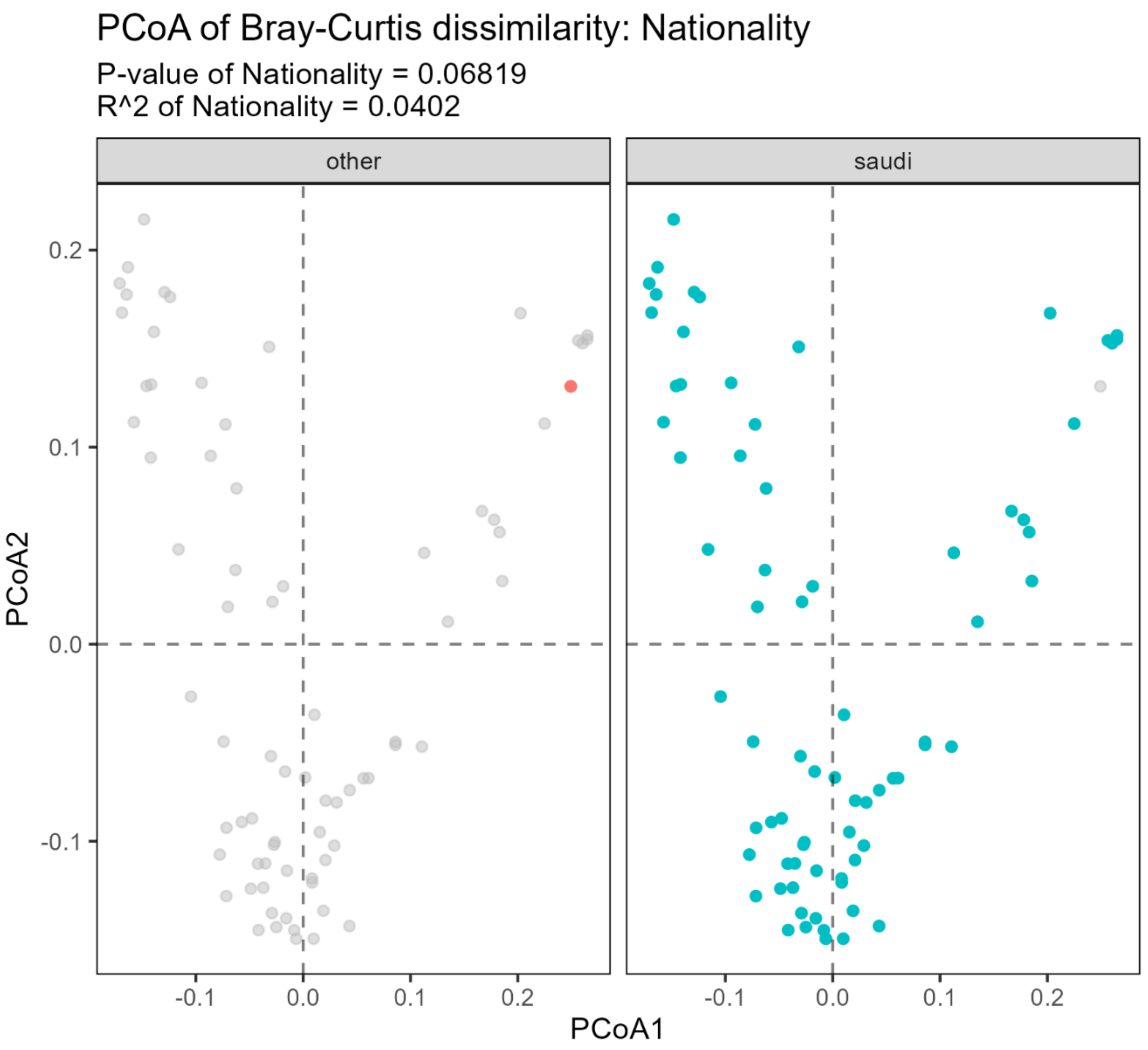
PCoA of Bray-Curtis dissimilarity: Nationality
P-value of Nationality = 0.06819
R^2 of Nationality = 0.0402
other
saudi
PCoA2
0.2
0.1
0.0
-0.1
-0.1
0.0
0.1
0.2
PCoA1

Fig S6.8B

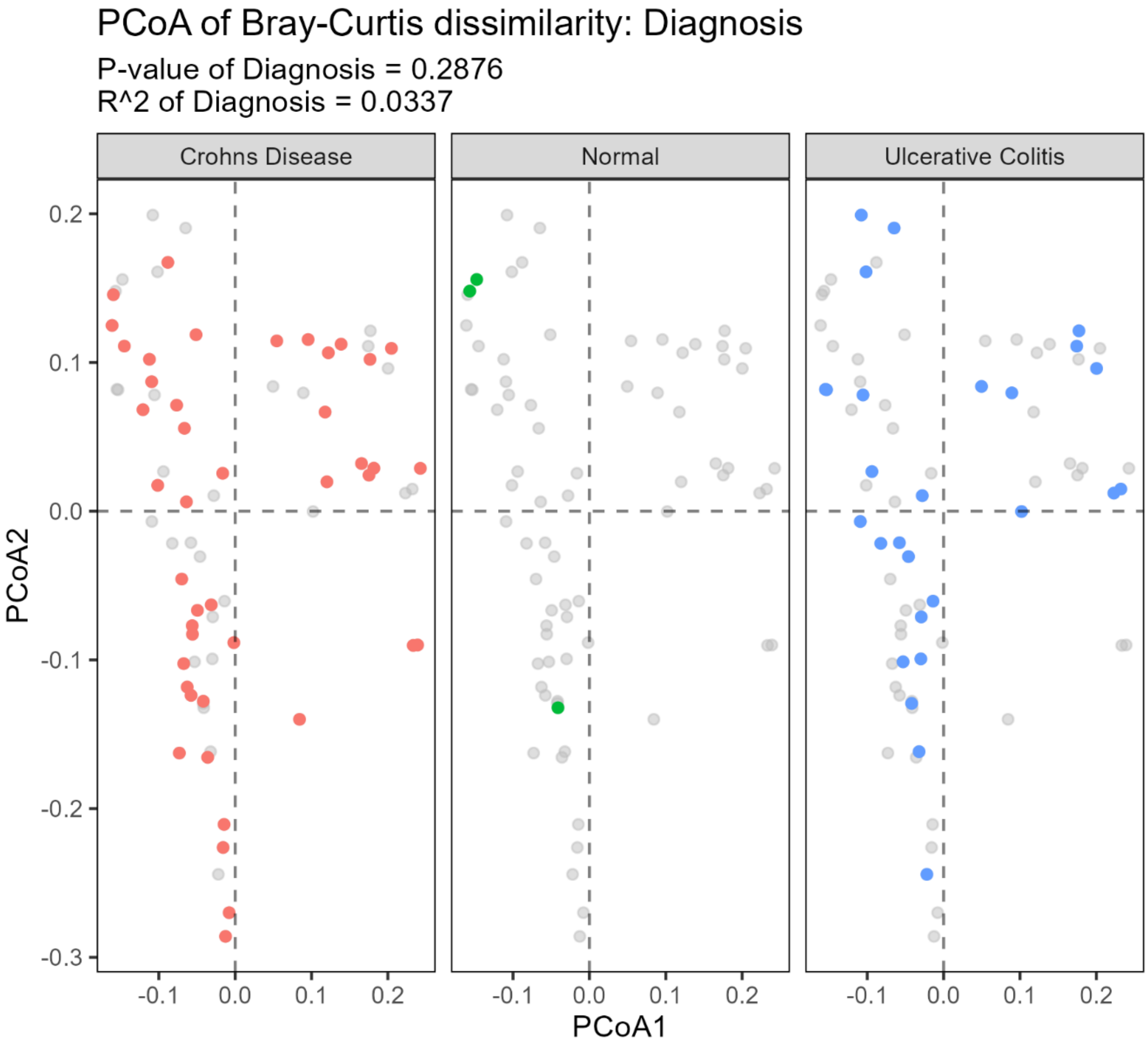
PCoA of Bray-Curtis dissimilarity: Diagnosis
P-value of Diagnosis = 0.2876
R^2 of Diagnosis = 0.0337
Crohns Disease
Normal
Ulcerative Colitis
PCoA2
0.2
0.1
0.0
-0.1
-0.2
-0.3
-0.1
0.0
0.1
0.2
PCoA1

Fig S6.9B

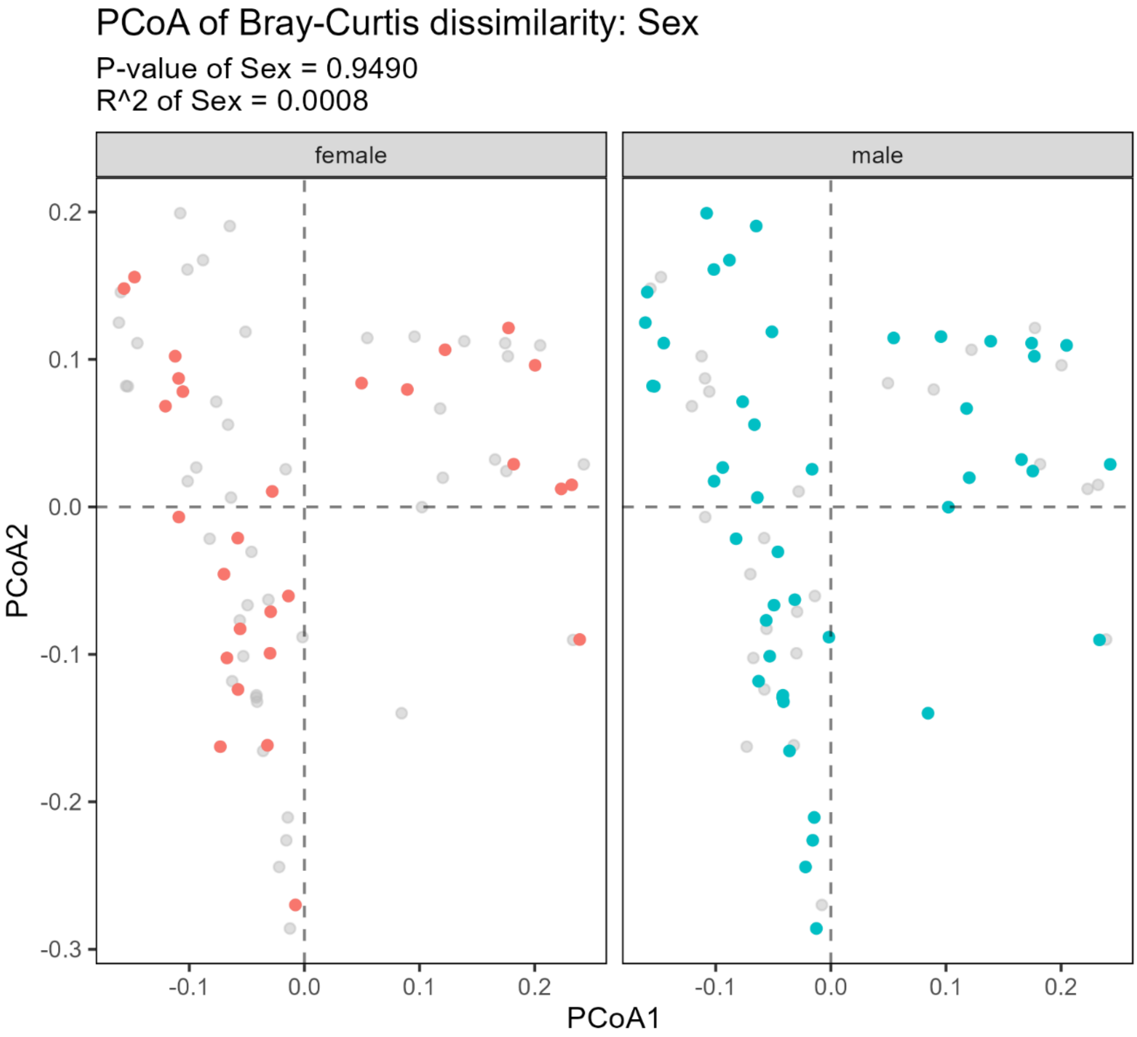
PCoA of Bray-Curtis dissimilarity: Sex
P-value of Sex = 0.9490
R^2 of Sex = 0.0008
female
male
0.2
0.1
0.0
-0.1
-0.2
-0.3
-0.1
0.0
0.1
0.2
PCoA2
PCoA1

Fig S6.10B

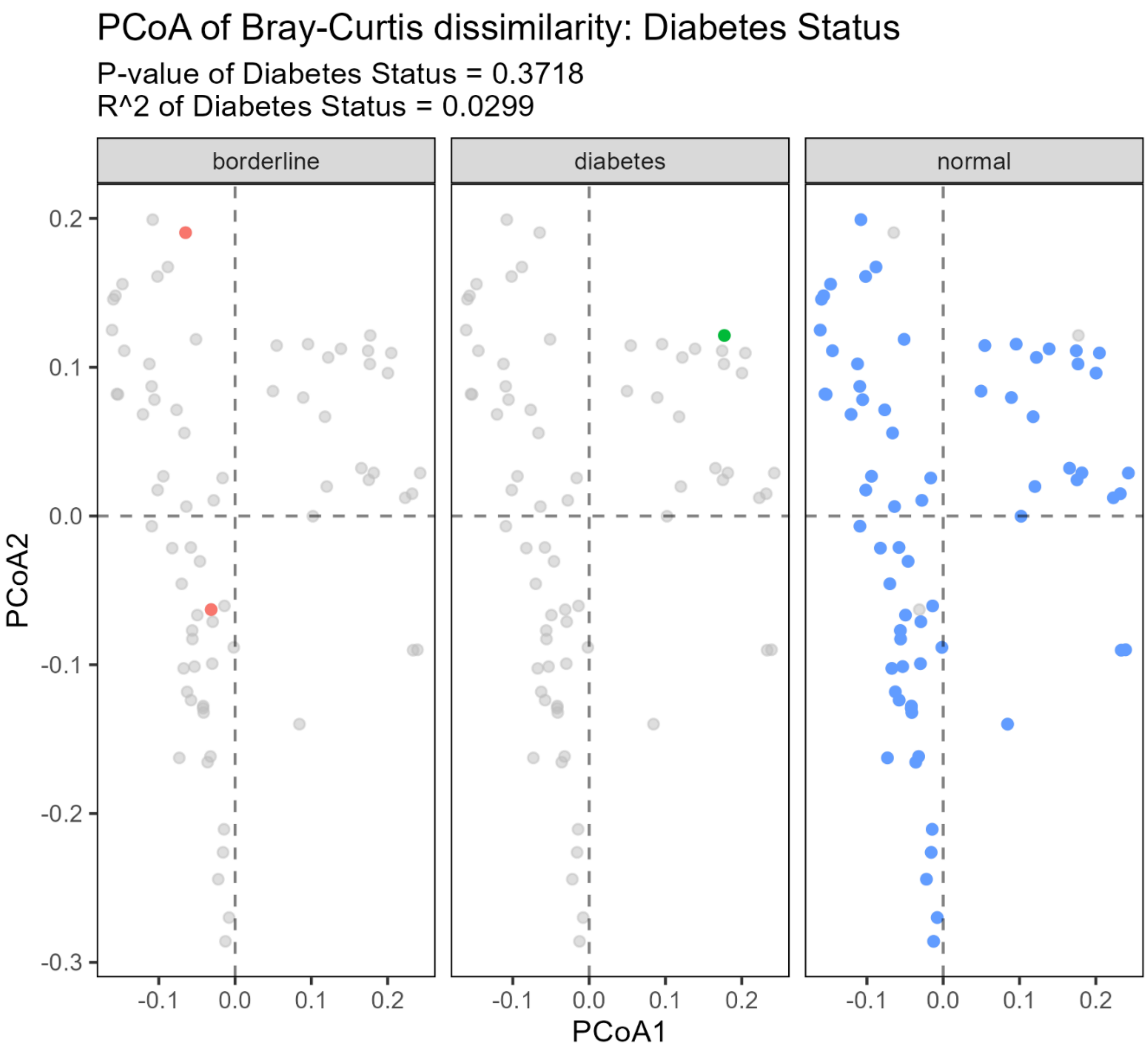
PCoA of Bray-Curtis dissimilarity: Diabetes Status
P-value of Diabetes Status = 0.3718
R^2 of Diabetes Status = 0.0299
borderline
diabetes
normal
0.2
0.1
0.0
-0.1
-0.2
-0.3
PCoA2
-0.1
0.0
0.1
0.2
PCoA1

Fig S6.11B

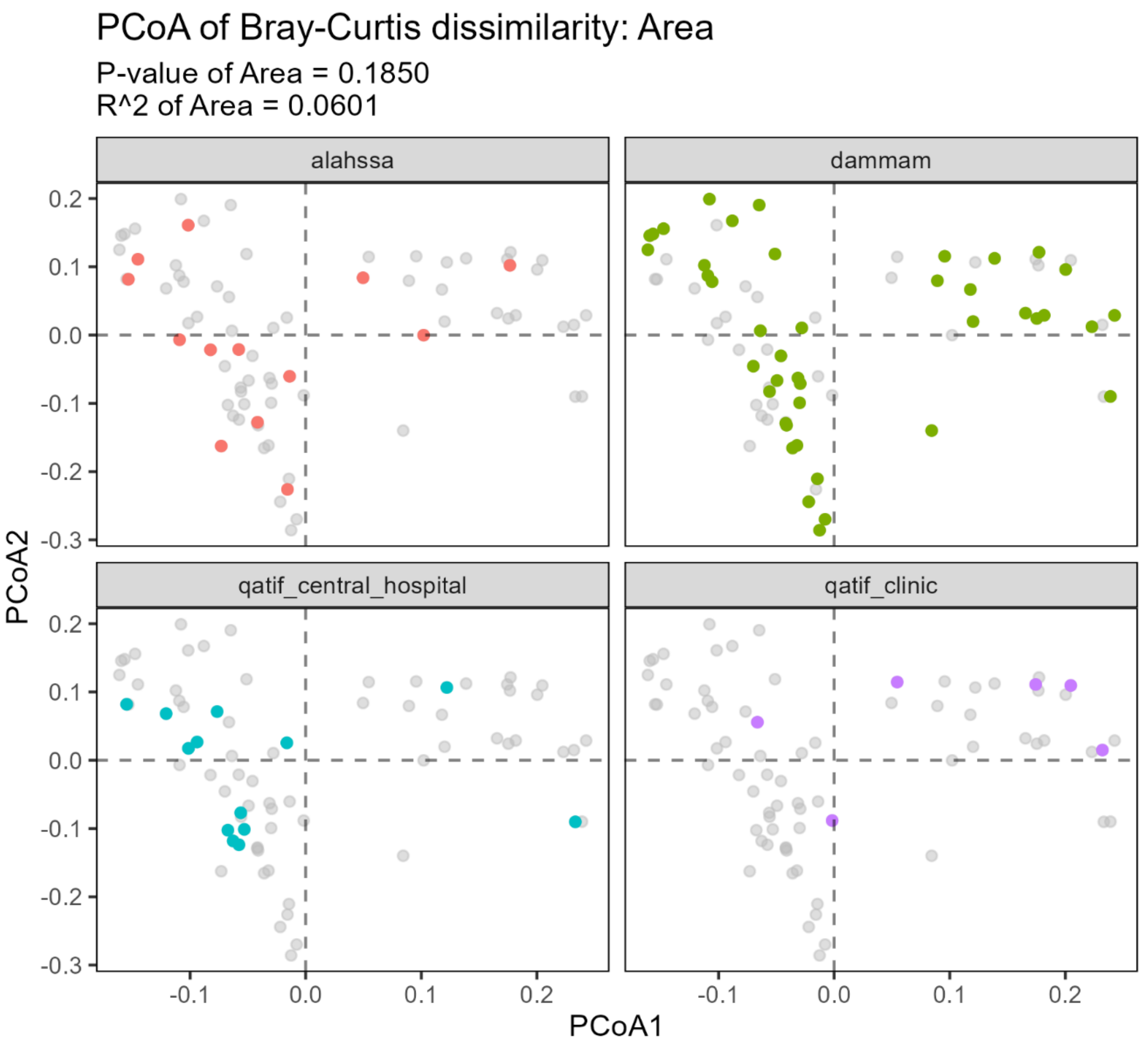

PCoA of Bray-Curtis dissimilarity: Area
P-value of Area = 0.1850
R^2 of Area = 0.0601
alahssa
dammam
qatif_central_hospital
qatif_clinic
PCoA2
PCoA1
0.2
0.1
0.0
-0.1
-0.2
-0.3
-0.1
0.0
0.1
0.2

Fig S6.12B

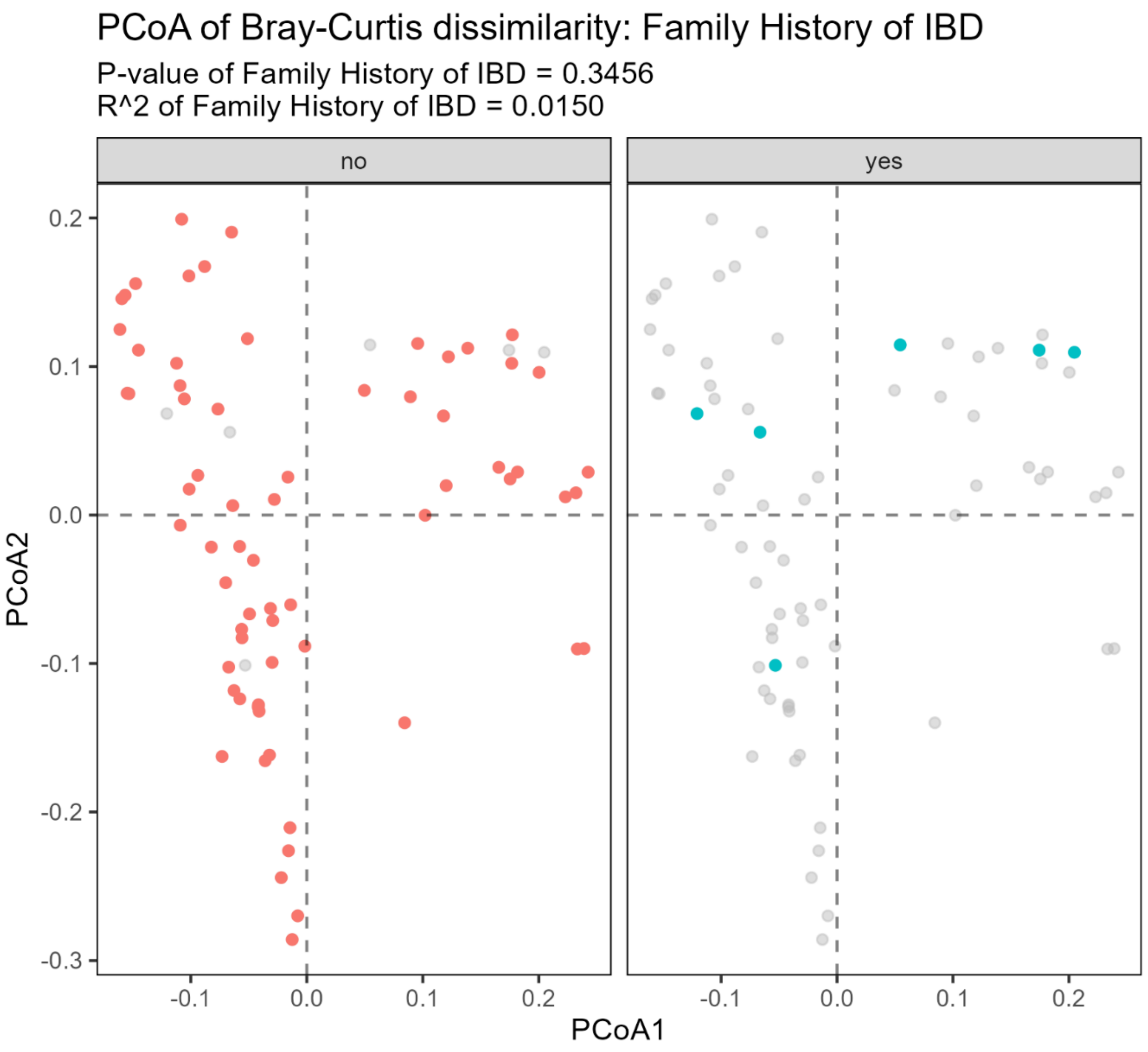
PCoA of Bray-Curtis dissimilarity: Family History of IBD
P-value of Family History of IBD = 0.3456
R^2 of Family History of IBD = 0.0150
no
yes
0.2
0.1
0.0
-0.1
-0.2
-0.3
PCoA2
-0.1
0.0
0.1
0.2
PCoA1

Fig S6.13B

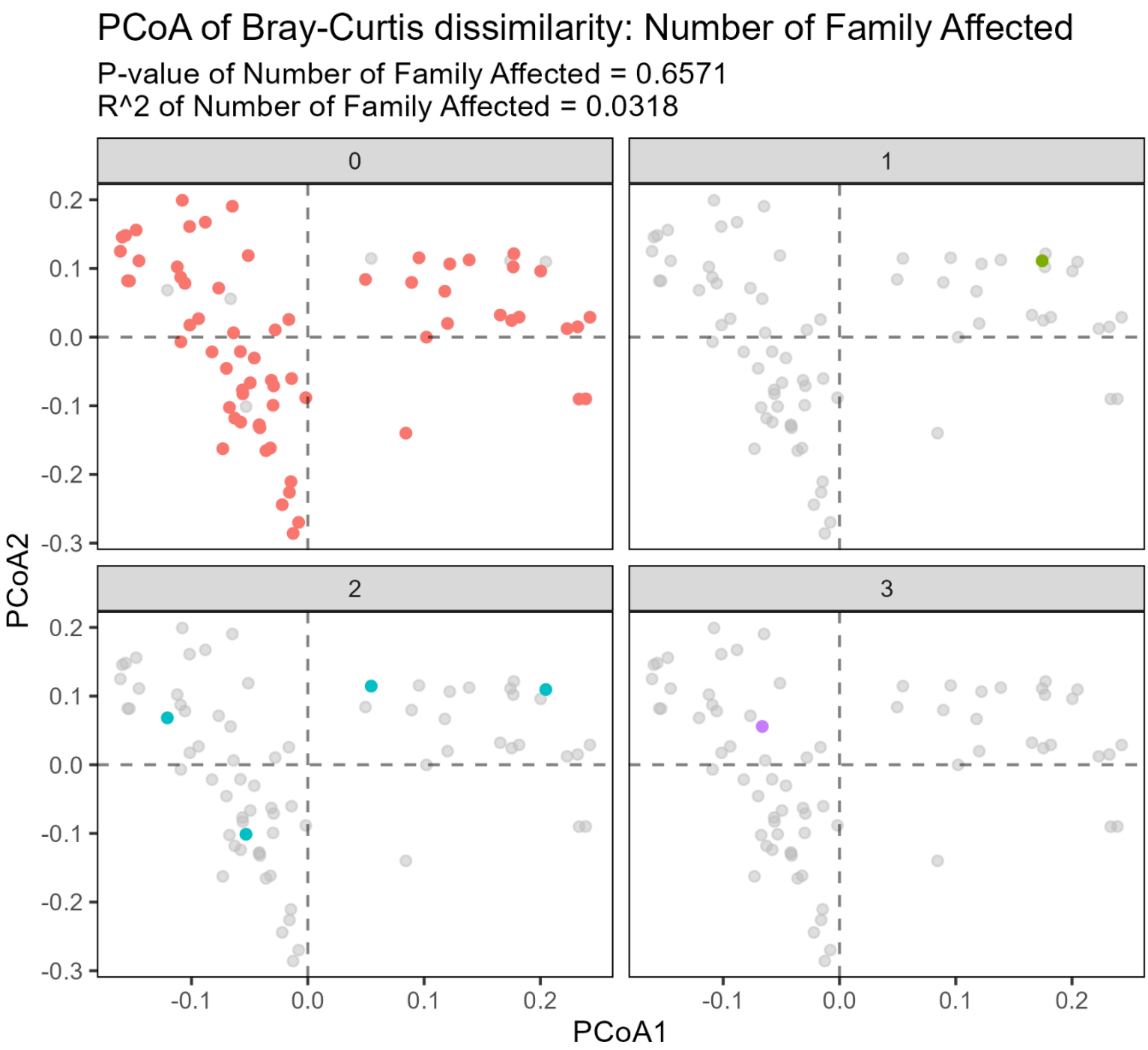
PCoA of Bray-Curtis dissimilarity: Number of Family Affected
P-value of Number of Family Affected = 0.6571
R^2 of Number of Family Affected = 0.0318
0
1
2
3
PCoA2
PCoA1
0.2
0.1
0.0
-0.1
-0.2
-0.3
-0.1
0.0
0.1
0.2

Fig S6.14B

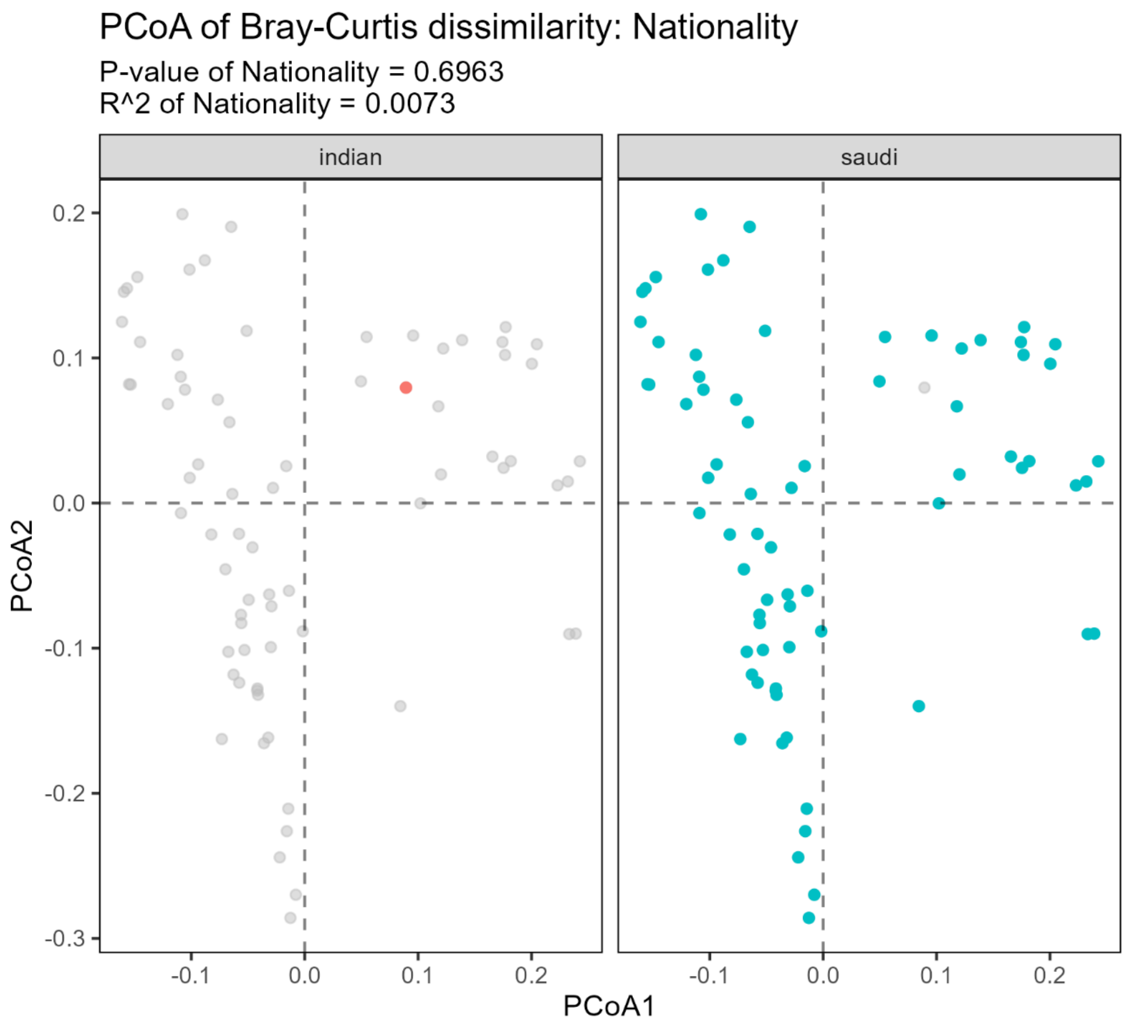
PCoA of Bray-Curtis dissimilarity: Nationality
P-value of Nationality = 0.6963
R^2 of Nationality = 0.0073
indian
saudi
PCoA2
PCoA1
0.2
0.1
0.0
-0.1
-0.2
-0.3
-0.1
0.0
0.1
0.2

Fig S6.15C

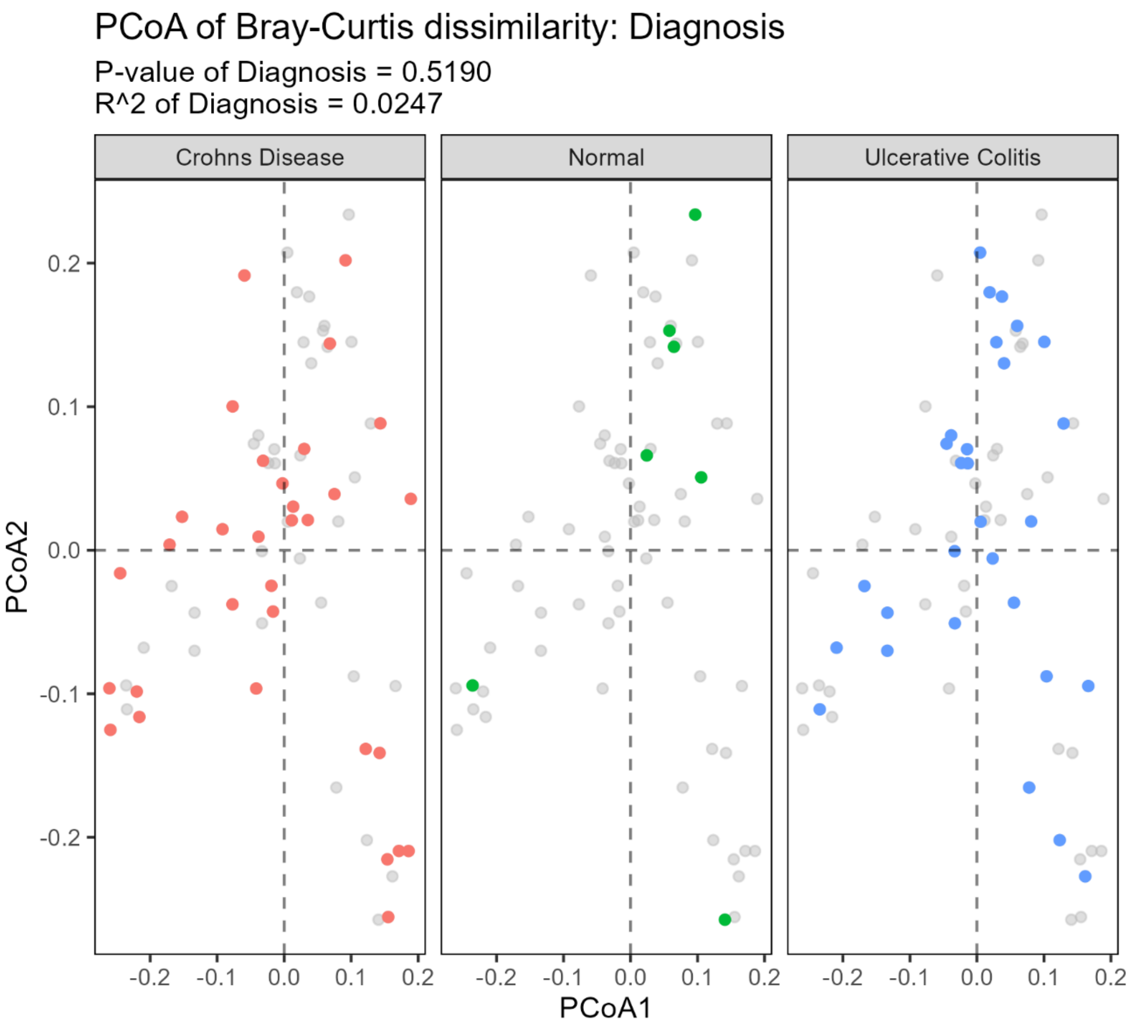
PCoA of Bray-Curtis dissimilarity: Diagnosis
P-value of Diagnosis = 0.5190
R^2 of Diagnosis = 0.0247
Crohns Disease
Normal
Ulcerative Colitis
0.2
0.1
0.0
-0.1
-0.2
PCoA2
-0.2
-0.1
0.0
0.1
0.2
PCoA1

Fig S6.16C

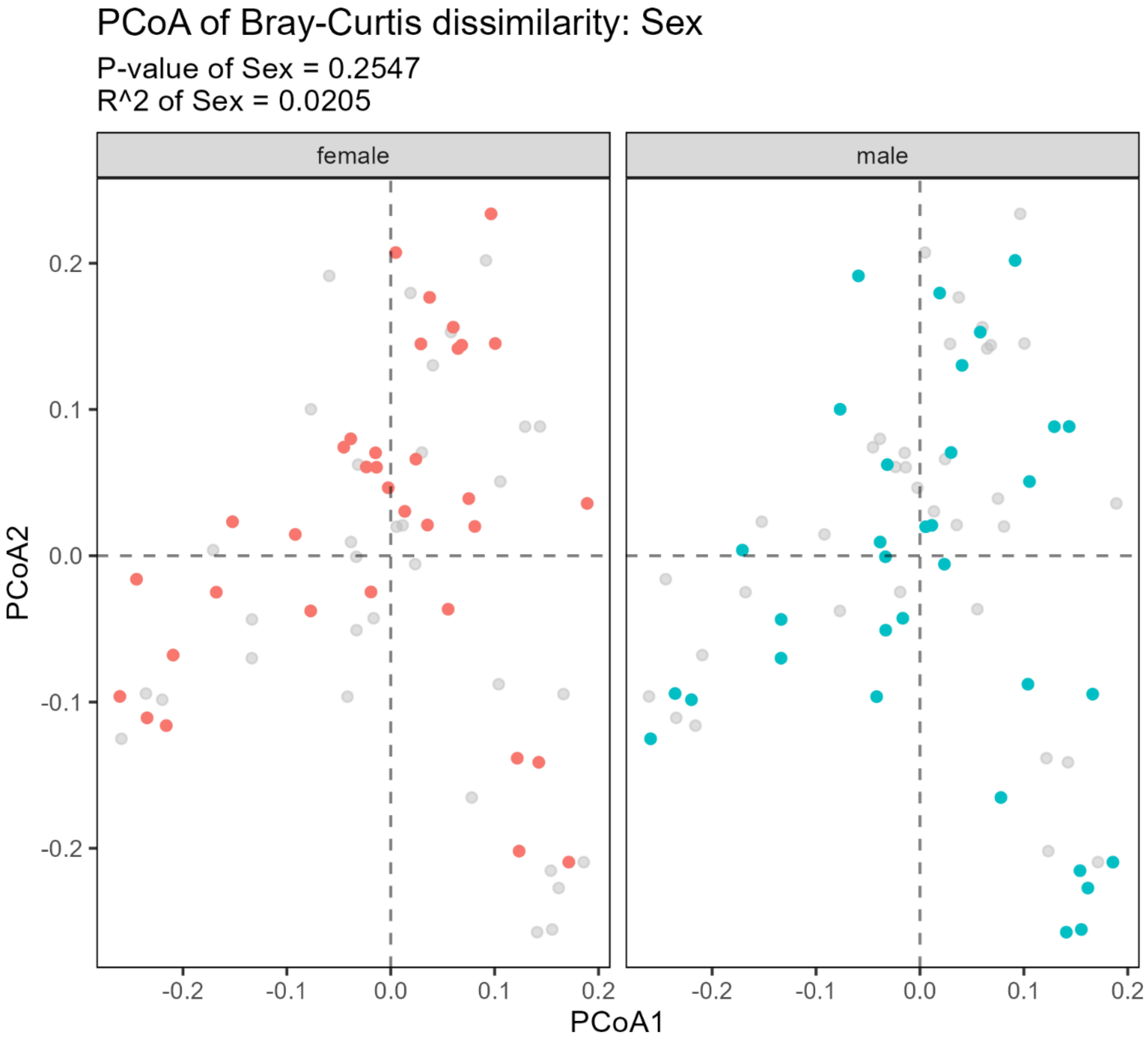
PCoA of Bray-Curtis dissimilarity: Sex
P-value of Sex = 0.2547
R^2 of Sex = 0.0205
female
male
0.2
0.1
0.0
-0.1
-0.2
PCoA2
-0.2
-0.1
0.0
0.1
0.2
PCoA1

Fig S6.17C

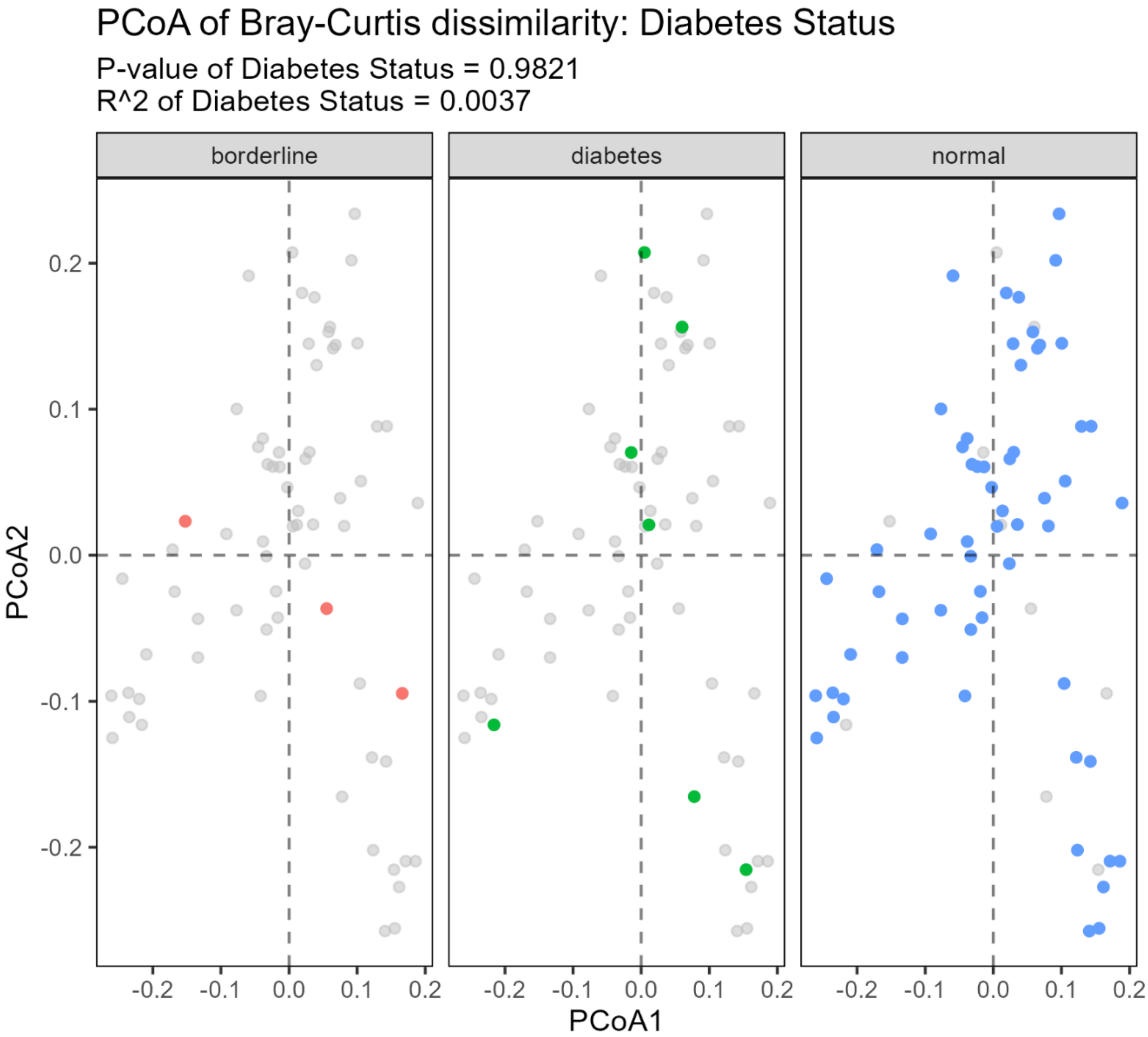

PCoA of Bray-Curtis dissimilarity: Diabetes Status
P-value of Diabetes Status = 0.9821
R^2 of Diabetes Status = 0.0037
borderline
diabetes
normal
PCoA2
PCoA1
0.2
0.1
0.0
-0.1
-0.2
-0.2 -0.1 0.0 0.1 0.2

Fig S6.18C

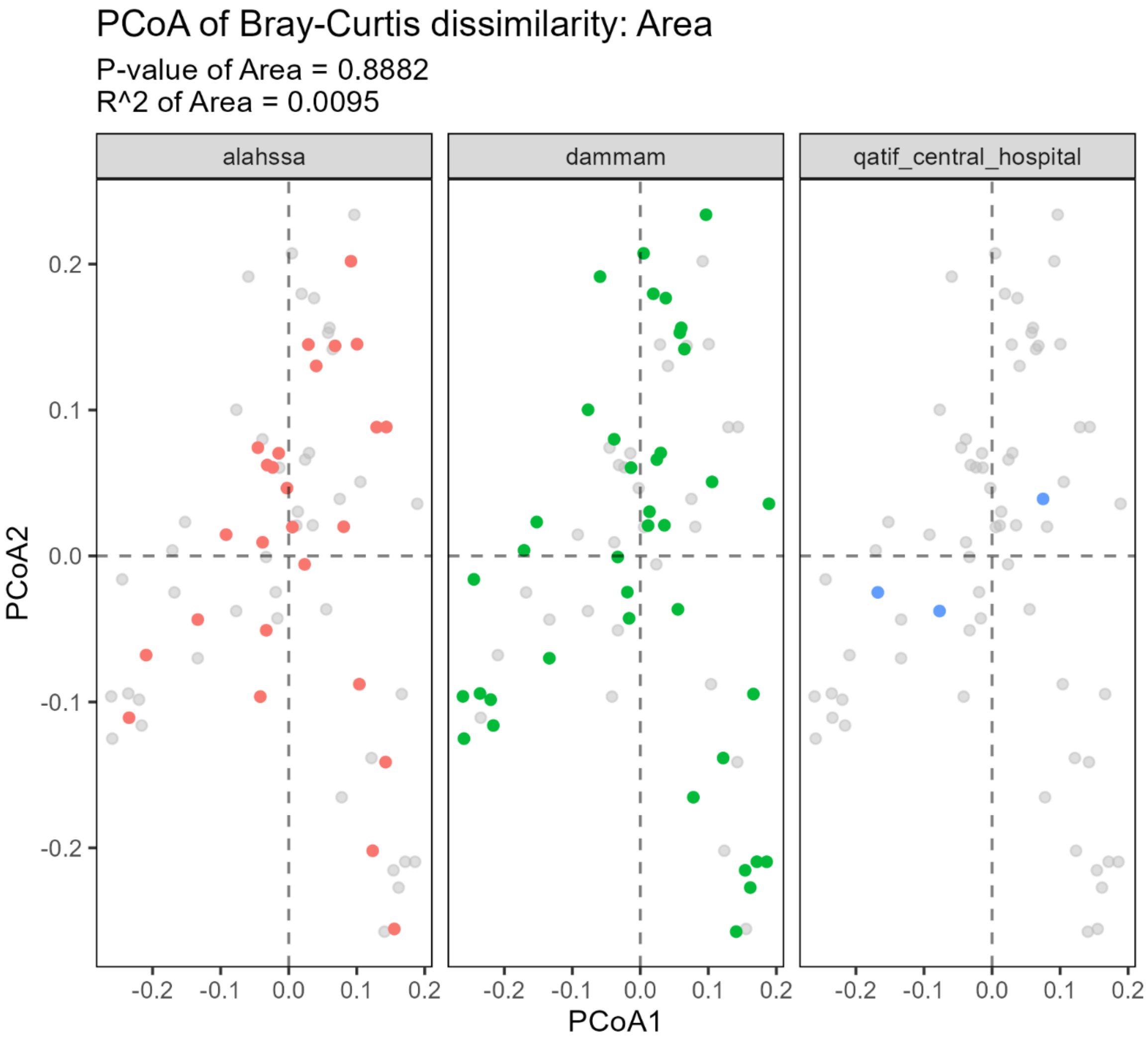

PCoA of Bray-Curtis dissimilarity: Area
P-value of Area = 0.8882
R^2 of Area = 0.0095
alahssa
dammam
qatif_central_hospital
PCoA2
0.2
0.1
0.0
-0.1
-0.2
-0.2
-0.1
0.0
0.1
0.2
PCoA1

Fig S6.19C

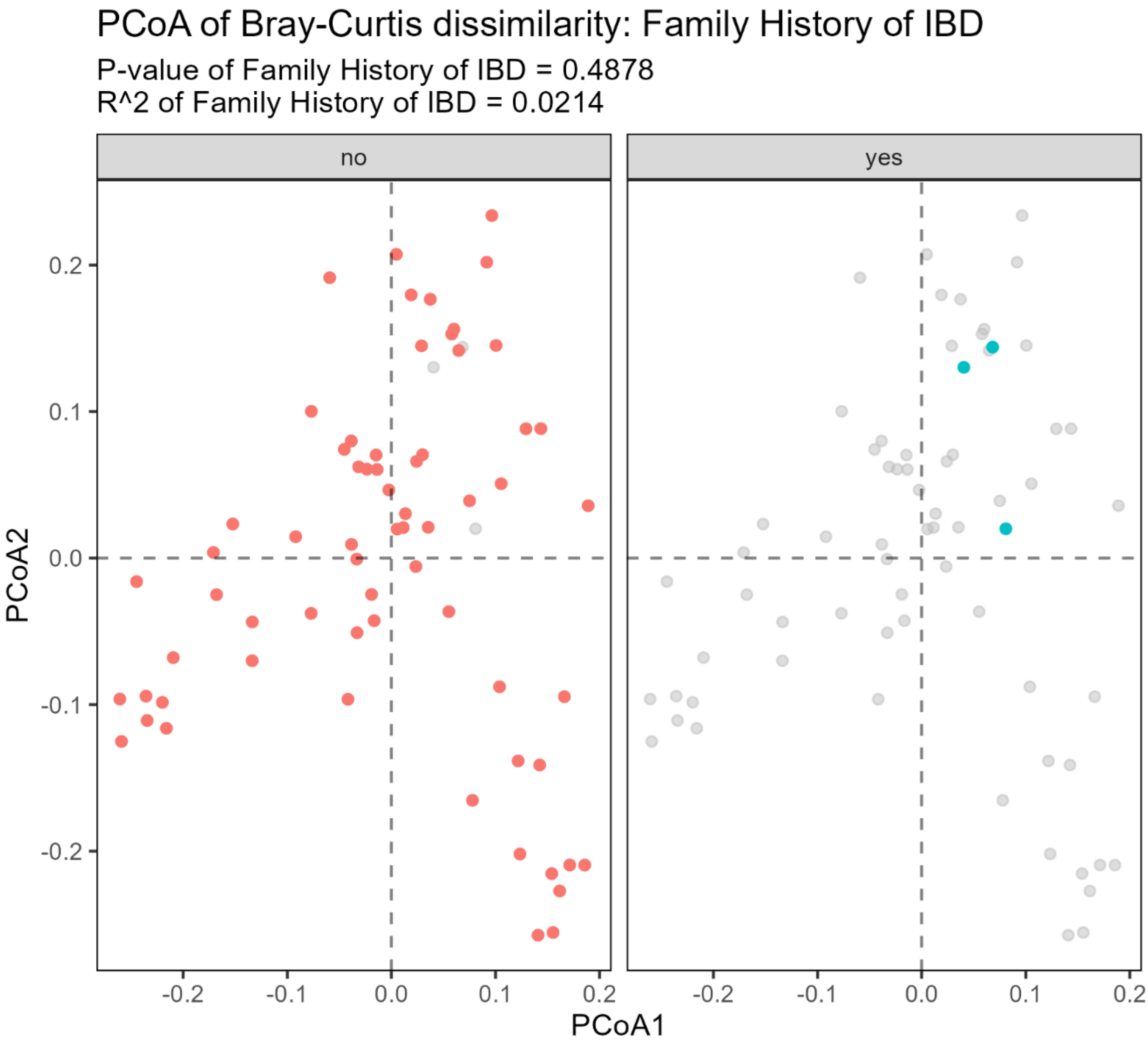

PCoA of Bray-Curtis dissimilarity: Family History of IBD
P-value of Family History of IBD = 0.4878
R^2 of Family History of IBD = 0.0214
no
yes
PCoA2
PCoA1
0.2
0.1
0.0
-0.1
-0.2
-0.2
-0.1
0.0
0.1
0.2

Fig S6.20C

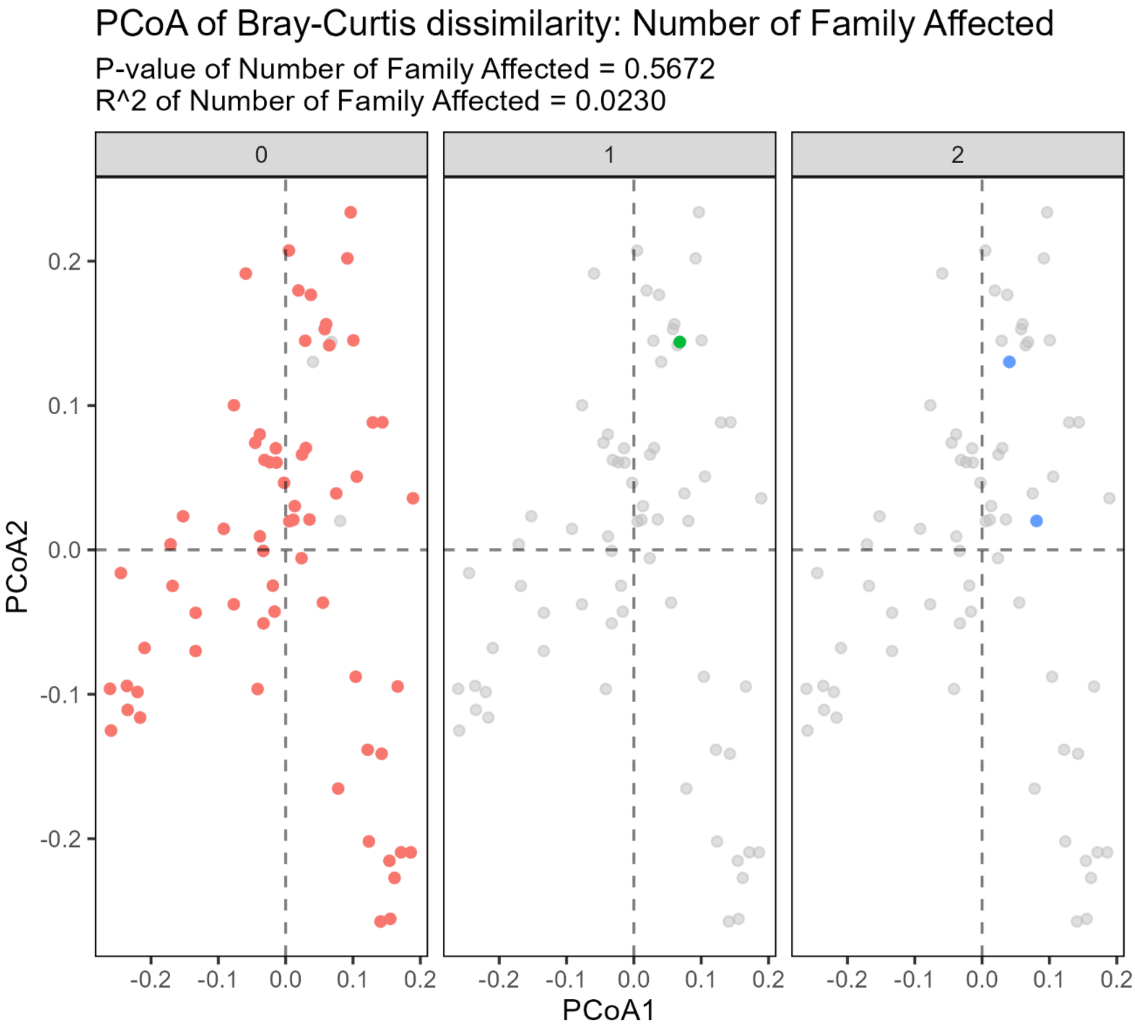
PCoA of Bray-Curtis dissimilarity: Number of Family Affected
P-value of Number of Family Affected = 0.5672
R^2 of Number of Family Affected = 0.0230
0
1
2
PCoA2
0.2
0.1
0.0
-0.1
-0.2
-0.2
-0.1
0.0
0.1
0.2
PCoA1

Fig S6.21C

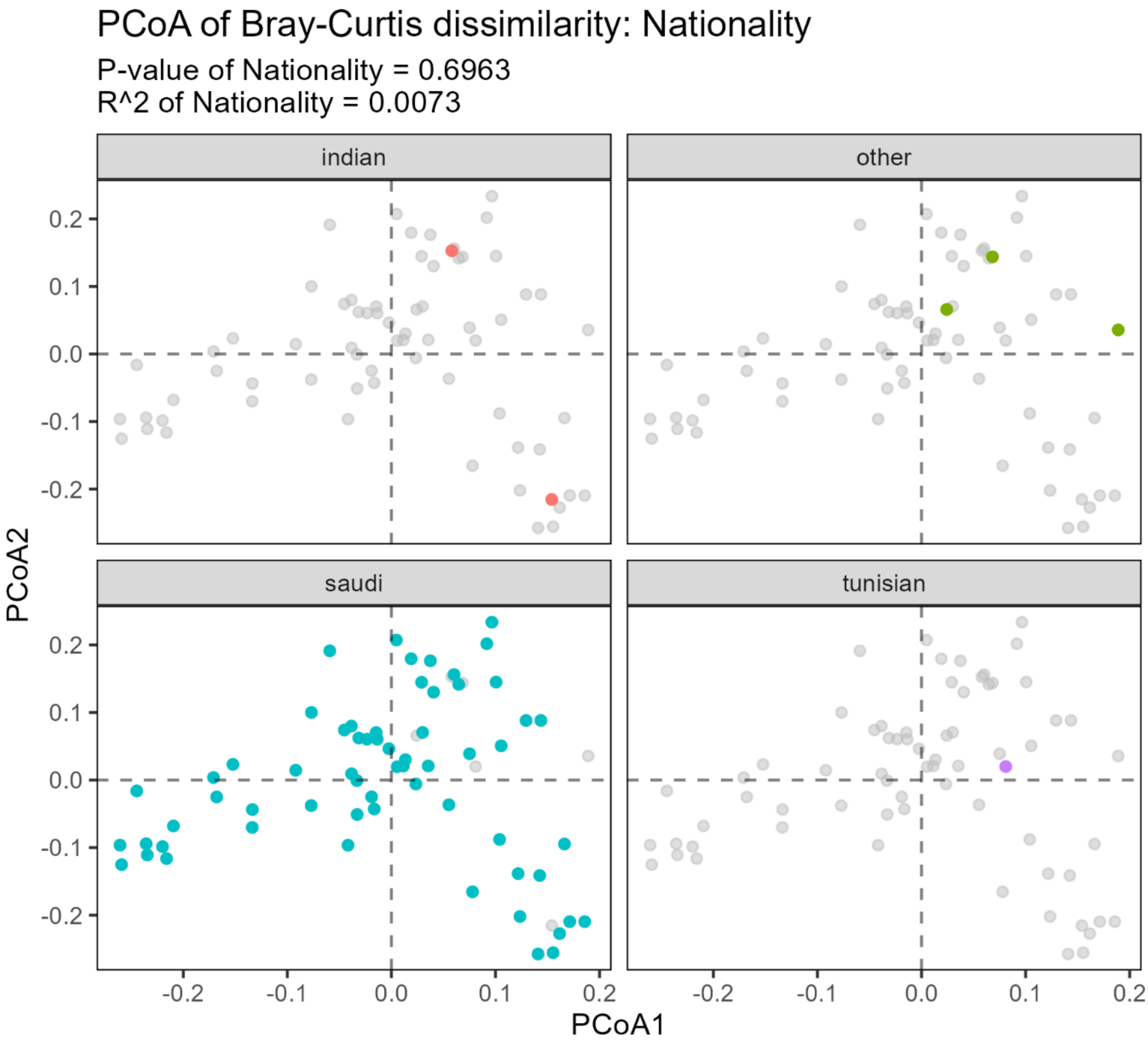

PCoA of Bray-Curtis dissimilarity: Nationality
P-value of Nationality = 0.6963
R^2 of Nationality = 0.0073
indian
other
saudi
tunisian
PCoA2
PCoA1
0.2
0.1
0.0
-0.1
-0.2
