# Supplementary material for: Gut microbiota analyses of inflammatory bowel diseases from a representative Saudi population
Source: BMC Gastroenterol. 2023 Jul 28;23:258. doi: 10.1186/s12876-023-02904-2 (PMC10375692; doi:10.1186/s12876-023-02904-2)
Supplement: Supplementary file 8 — Supplementary Material 8 [file 12876_2023_2904_MOESM8_ESM.pdf]

Additional File 8: Fig. S7. Evaluated the proportion of uncharacterized OTUs at each taxonomic level. Bray-Curtis PCoA scatterplots of dissimilarity on principal coordinates axes 1 and 2. The analysis done by sorting each patient into one of three

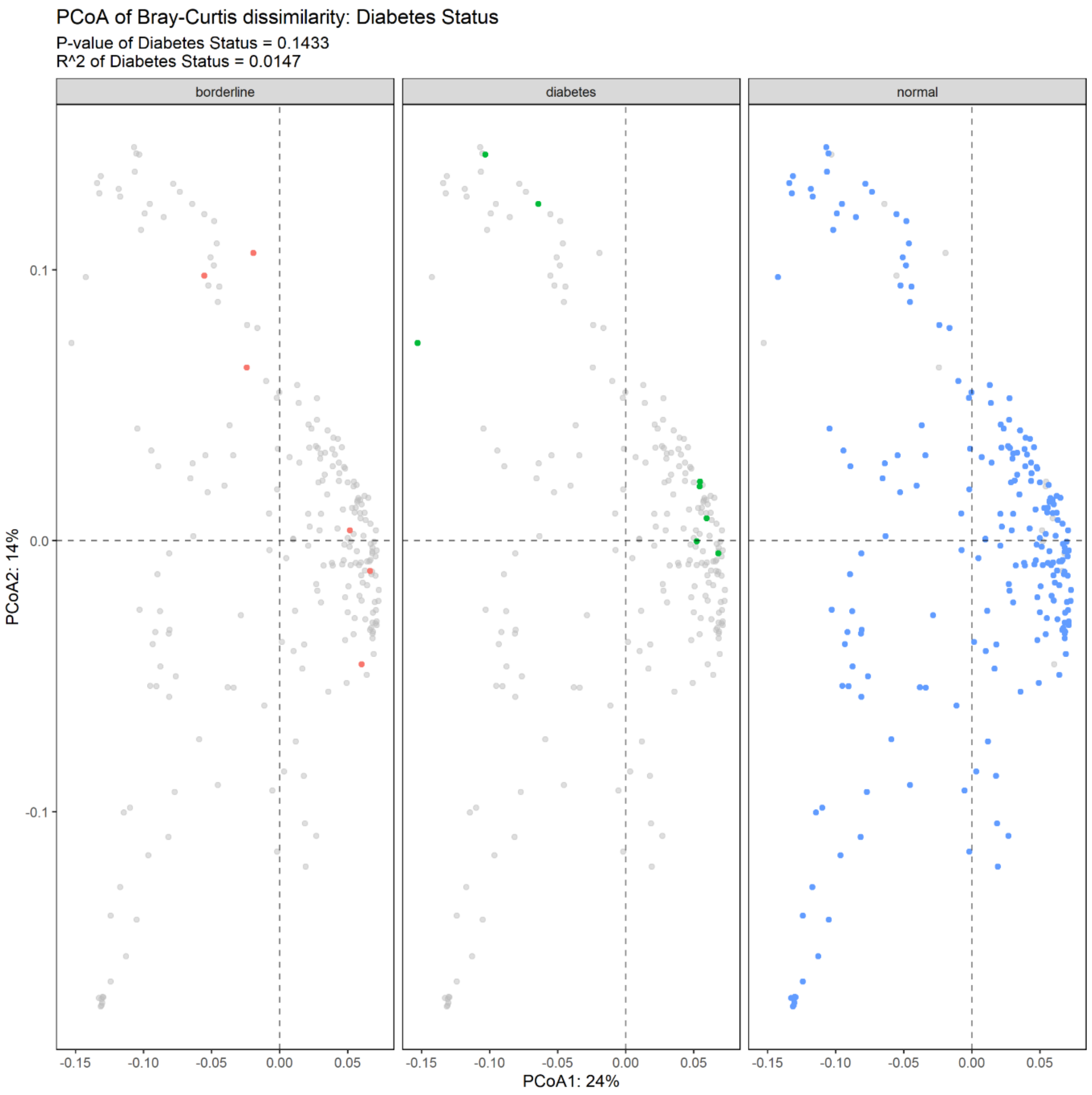

bins based on their age (0-27 years, 28-36 years, and >36 years).
