# Supplementary material for: Gut microbiota analyses of inflammatory bowel diseases from a representative Saudi population
Source: BMC Gastroenterol. 2023 Jul 28;23:258. doi: 10.1186/s12876-023-02904-2 (PMC10375692; doi:10.1186/s12876-023-02904-2)

Additional File 9: Fig S8. Determination of which variables shows a statistically significant effect on beta diversity. Bray-Curtis PCoA scatterplots of dissimilarity on principal coordinates axes 1 and 2. The analysis done by sorting each patient into one of three bins based on their age (0-27 years, 28-36 years, and >36 years).

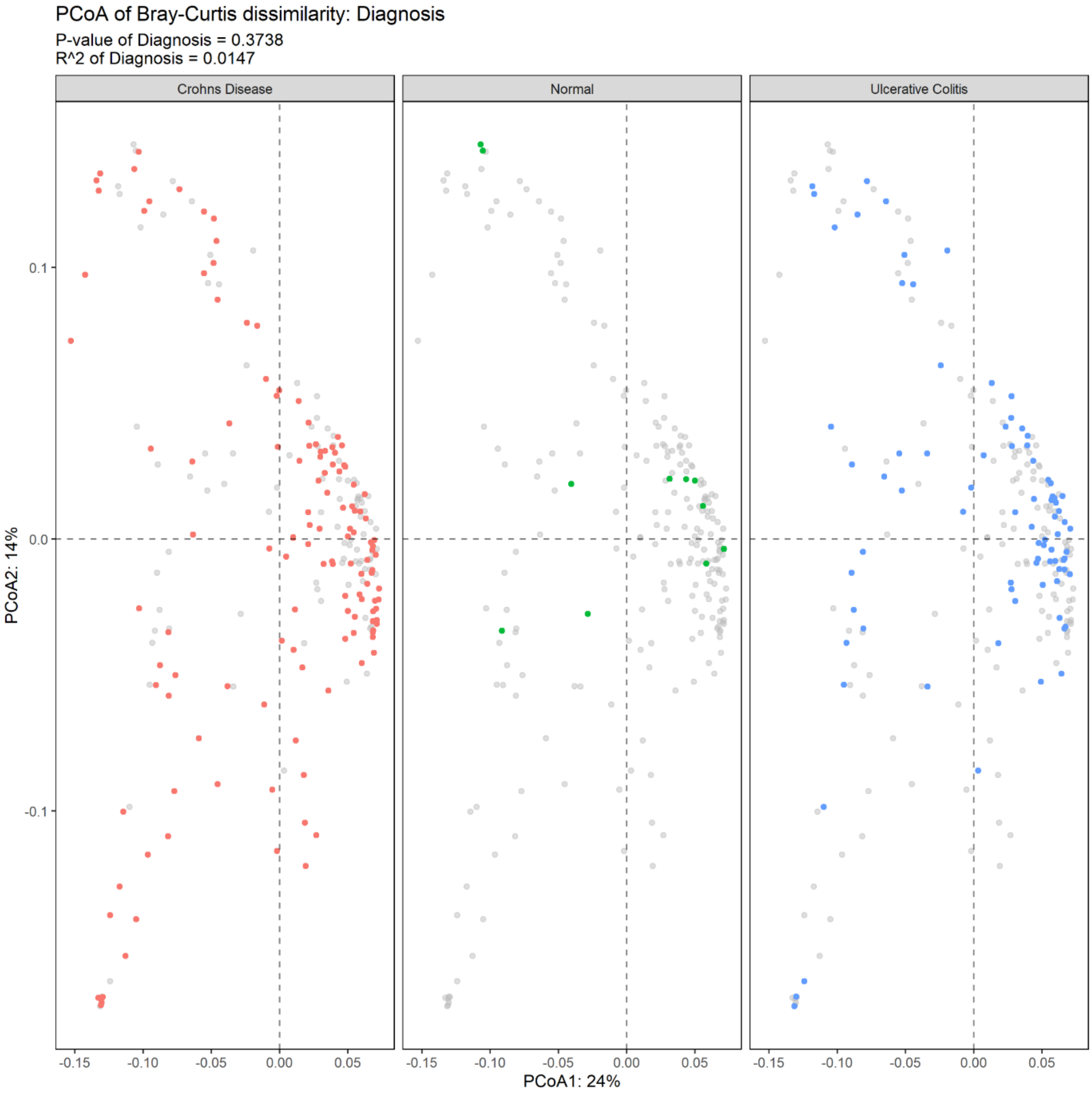

Supplement: Supplementary file 9 — Supplementary Material 9 [file 12876_2023_2904_MOESM9_ESM.pdf]
