# Supplementary material for: Gut microbiota analyses of inflammatory bowel diseases from a representative Saudi population
Source: BMC Gastroenterol. 2023 Jul 28;23:258. doi: 10.1186/s12876-023-02904-2 (PMC10375692; doi:10.1186/s12876-023-02904-2)

Additional File 10: Fig S9. Determination of which variables shows a statistically significant effect on beta diversity. Bray-Curtis PCoA scatterplots of dissimilarity on principal coordinates axes 1 and 2.

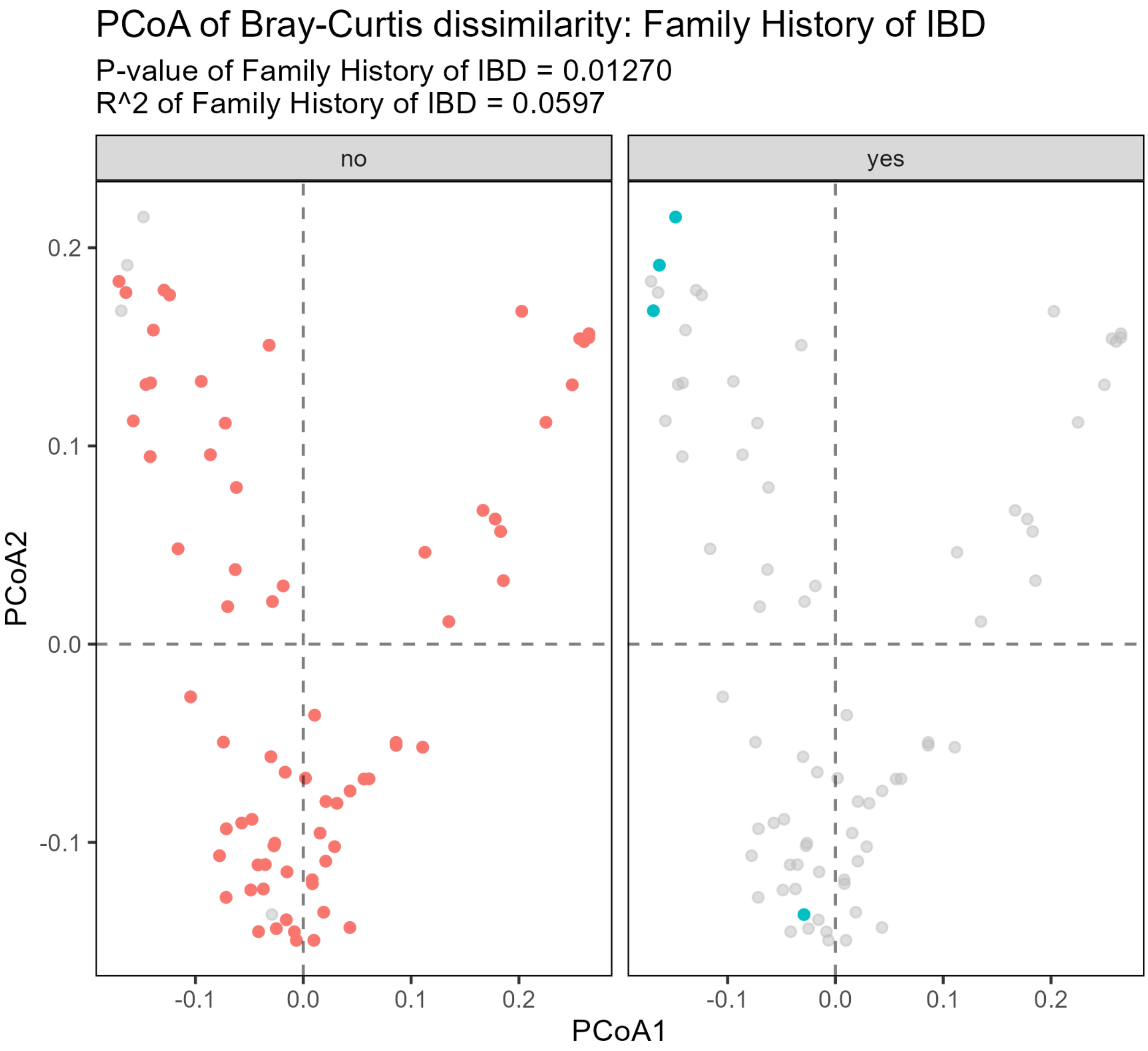

Supplement: Supplementary file 10 — Supplementary Material 10 [file 12876_2023_2904_MOESM10_ESM.pdf]
