# Supplementary figures and images for: Gut microbiota analyses of inflammatory bowel diseases from a representative Saudi population
Source: BMC Gastroenterol. 2023 Jul 28;23:258. doi: 10.1186/s12876-023-02904-2 (PMC10375692; doi:10.1186/s12876-023-02904-2)

Differential abundance at the species level of ulcerative colitis compared to normal.

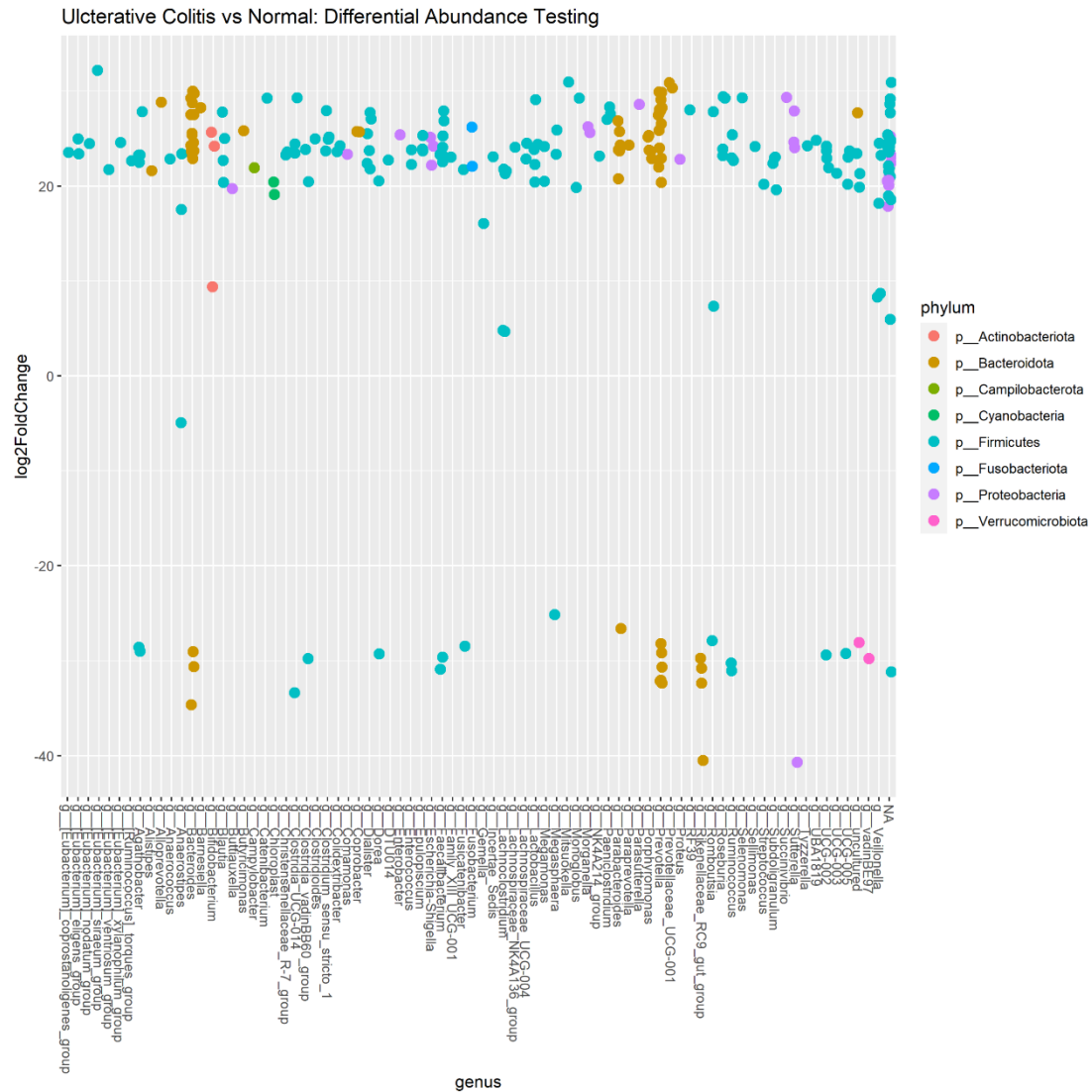

Supplement: Supplementary file 12 — Supplementary Material 12 [file 12876_2023_2904_MOESM12_ESM.pdf]

Differential abundance at the species level of Crohn's disease compared to normal.

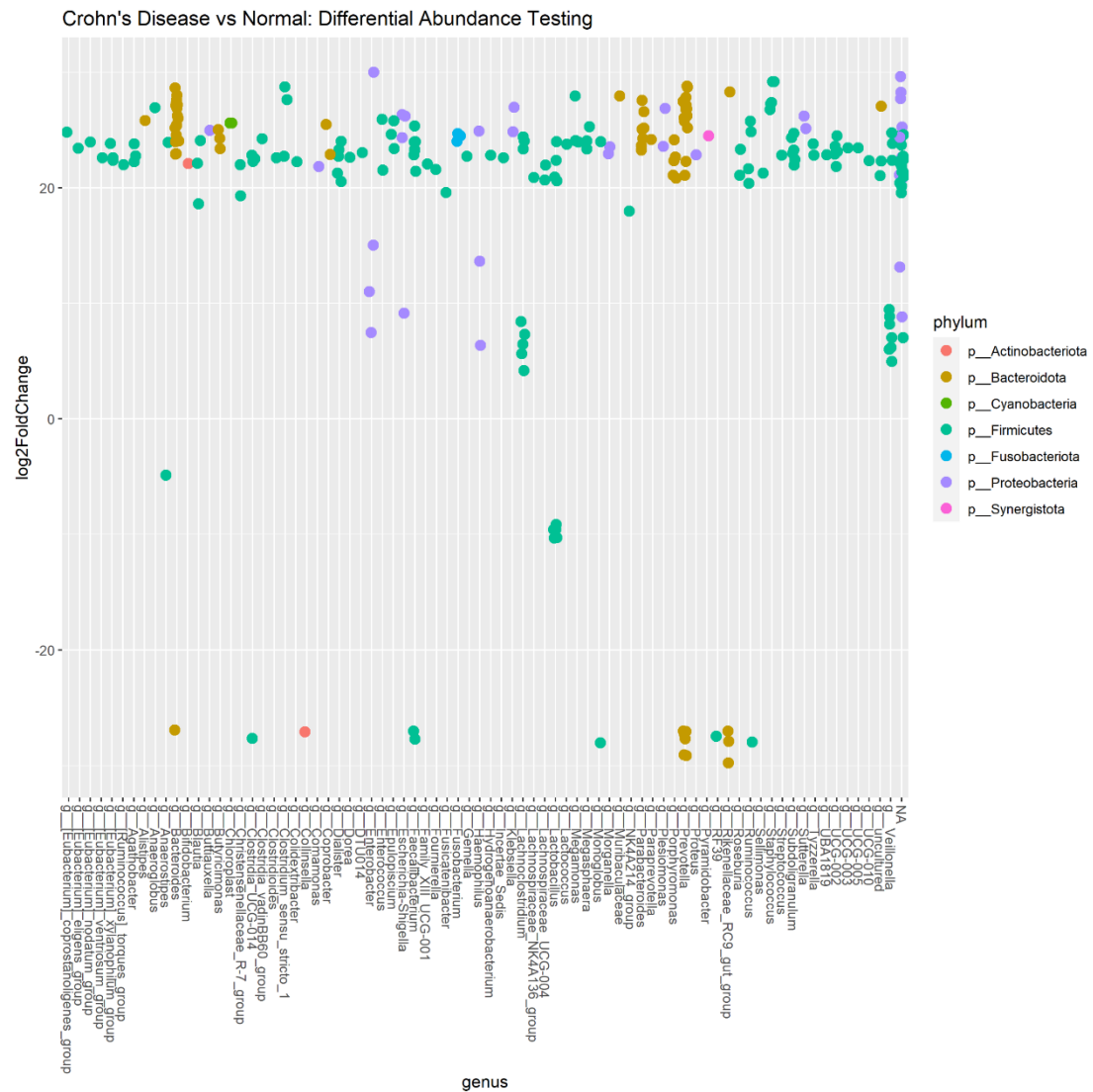

Supplement: Supplementary file 13 — Supplementary Material 13 [file 12876_2023_2904_MOESM13_ESM.pdf]
