# Supplementary material for: Gut microbiota analyses of inflammatory bowel diseases from a representative Saudi population
Source: BMC Gastroenterol. 2023 Jul 28;23:258. doi: 10.1186/s12876-023-02904-2 (PMC10375692; doi:10.1186/s12876-023-02904-2)
Supplement: Supplementary file 14 — Supplementary Material 14 [file 12876_2023_2904_MOESM14_ESM.pdf]

Additional File 14: Supplementary Table S2: Species Level Differential Abundance Testing. Species Level Differential Abundance Testing for the Top 20 upregulated (top) and downregulated (bottom) for Ulcerative Colitis (UC) versus normal with Operational Taxonomic Unit (OTU) outputs along with the significance values for each of the top 20 genus-species levels.

OTU	log2FoldChange	Adjusted P-Value	Genus	Species
otu583041	24.784	6.53E-26	Blautia	Blautia_hansenii
otu322337	24.588	4.52E-15	Clostridium_sensu_stricto_1	Clostridium_perfringens
otu335922	24.891	4.16E-13	Ruminococcus	Clostridiaceae_bacterium
otu111341	25.398	7.76E-12	Dialister	Dialister_propionicifaciens
otu443743	23.862	2.74E-10	Dialister	Dialister_invisus
otu447833	24.075	2.96E-10	Enterococcus	Enterococcus_durans
otu191757	24.419	4.28E-10	Tyzzerella	Tyzzerella_sp.
otu449365	20.526	8.09E-10	Chloroplast	Punica_granatum
otu253552	21.282	8.91E-10	Lachnoclostridium	bacterium_NLAE-zl-H31
otu846127	24.134	9.70E-10	Parabacteroides	Parabacteroides_johnsonii
otu445760	21.731	1.47E-09	Dorea	Dorea_formicigenerans
otu70276	25.689	1.87E-09	Clostridioides	Clostridioides_difficile
otu110587	25.490	2.54E-09	Dialister	Dialister_micraerophilus
otu316853	25.677	4.27E-09	Parabacteroides	Parabacteroides_johnsonii
otu113756	24.172	8.35E-09	Megamonas	Megamonas_funiformis
otu185316	25.556	9.99E-09	Bacteroides	Bacteroides_ovatus
otu789812	25.495	3.98E-08	Escherichia-Shigella	Escherichia_sp.
otu494919	23.648	5.31E-08	Porphyromonas	Porphyromonas_asaccharolytica
otu434222	28.065	1.43E-07	Prevotella	Prevotella_oris
otu738264	25.207	3.24E-07	Comamonas	Comamonas_kerstersii

OTU	log2FoldChange	Adjusted P-Value	Genus	Species
otu294573	-37.169	2.32E-06	Prevotella	Trichuris_trichiura
otu326482	-35.597	7.53E-06	Prevotella	Prevotella_copri
otu446847	-34.522	1.64E-05	Bacteroides	Bacteroides_stercoris
otu357901	-26.648	1.36E-04	Parabacteroides	Parabacteroides_goldsteinii
otu196388	-31.008	1.61E-04	Agathobacter	[Eubacterium]_rectale
otu258695	-30.614	1.98E-04	Bacteroides	Bacteroidaceae_bacterium
otu228081	-30.321	2.35E-04	Prevotella	Trichuris_trichiura
otu431164	-29.681	3.43E-04	vadinBE97	Bacteroidales_bacterium
otu180903	-29.293	4.26E-04	Dorea	Dorea_formicigenerans
otu209685	-29.077	4.64E-04	Bacteroides	Bacteroides_coprocola
otu300872	-29.084	4.64E-04	Prevotella	Trichuris_trichiura
otu195651	-28.489	6.34E-04	Agathobacter	[Eubacterium]_rectale
otu531121	-27.480	1.00E-03	Prevotella	Prevotella_copri
otu198071	-27.135	1.16E-03	Agathobacter	[Eubacterium]_rectale
otu210523	-25.576	1.24E-03	Prevotella	Prevotella_copri
otu188128	-4.581	1.28E-03	Anaerostipes	Anaerostipes_hadrus
otu839765	-26.927	1.28E-03	Bacteroides	Bacteroides_stercoris
otu612107	-26.825	1.33E-03	Paraprevotella	Paraprevotella_clara
otu329650	-26.348	1.69E-03	Prevotella	Prevotella_copri
otu520056	-26.119	1.86E-03	Prevotella	Prevotella_copri
