# Supplementary material for: Gut microbiota analyses of inflammatory bowel diseases from a representative Saudi population
Source: BMC Gastroenterol. 2023 Jul 28;23:258. doi: 10.1186/s12876-023-02904-2 (PMC10375692; doi:10.1186/s12876-023-02904-2)
Supplement: Supplementary file 15 — Supplementary Material 15 [file 12876_2023_2904_MOESM15_ESM.pdf]

Additional File 15: Supplementary Table S3: Supplementary Table 3: **Species Level Differential Abundance Testing.** Species Level Differential Abundance Testing for the Top 20 upregulated (top) and downregulated (bottom) for Crohn's Disease (CD) versus normal with Operational Taxonomic Unit (OTU) outputs along with the significance values for each of the top 20 genus-species levels.

OTU	log2FoldChange	Adjusted P-Value	Genus	Species
otu583041	23.858	3.97E-25	g__Blautia	s__Blautia_hansenii
otu449365	25.701	8.06E-16	g__Chloroplast	s__Punica_granatum
otu322337	22.398	2.33E-13	g__Clostridium_sensu_stricto_1	s__Clostridium_perfringens
otu335922	24.316	2.49E-13	g__Ruminococcus	s__Clostridiaceae_bacterium
otu253552	23.299	2.78E-12	g__Lachnoclostridium	s__bacterium_NLAE-zl-H31
otu113756	27.923	3.31E-12	g__Megamonas	s__Megamonas_funiformis
otu445760	23.846	4.61E-12	g__Dorea	s__Dorea_formicigenerans
otu846127	24.979	5.52E-11	g__Parabacteroides	s__Parabacteroides_johnsonii
otu316853	27.503	6.72E-11	g__Parabacteroides	s__Parabacteroides_johnsonii
otu110587	26.531	1.34E-10	g__Dialister	s__Dialister_micraerophilus
otu191757	23.986	2.50E-10	g__Tyzzerella	s__Tyzzerella_sp.
otu789812	27.611	5.03E-10	g__Escherichia-Shigella	s__Escherichia_sp.
otu185316	25.377	4.99E-09	g__Bacteroides	s__Bacteroides_ovatus
otu358741	28.528	5.91E-09	g__Clostridium_sensu_stricto_1	s__Clostridium_butyricum
otu447833	21.790	6.09E-09	g__Enterococcus	s__Enterococcus_durans
otu443743	21.397	7.59E-09	g__Dialister	s__Dialister_invisus
otu494919	24.149	8.12E-09	g__Porphyromonas	s__Porphyromonas_asaccharolytica
otu525261	23.183	1.83E-08	g__Streptococcus	s__Streptococcus_lutetiensis
otu111341	20.411	2.60E-08	g__Dialister	s__Dialister_propionicifaciens
otu70276	23.320	2.63E-08	g__Clostridioides	s__Clostridioides_difficile

OTU	log2FoldChange	Adjusted P-Value	Genus	Species
otu553352	-9.600	2.09E-06	g__Lactobacillus	s__Lactobacillus_gasseri
otu578653	-9.151	2.17E-04	g__Lactobacillus	s__Lactobacillus_acidophilus
otu294573	-28.994	2.93E-04	g__Prevotella	s__Trichuris_trichiura
otu188128	-4.873	3.62E-04	g__Anaerostipes	s__Anaerostipes_hadrus
otu864573	-9.584	4.88E-04	g__Lactobacillus	s__Lactobacillus_gasseri
otu326482	-27.532	6.63E-04	g__Prevotella	s__Prevotella_copri
otu364584	-27.287	7.44E-04	g__Prevotella	s__Prevotella_stercorea
otu228081	-27.209	7.73E-04	g__Prevotella	s__Trichuris_trichiura
otu313977	-27.103	8.11E-04	g__Collinsella	s__Collinsella_aerofaciens
otu213700	-26.938	8.68E-04	g__Bacteroides	s__Bacteroides_vulgatus
otu446847	-26.486	1.08E-03	g__Bacteroides	s__Bacteroides_stercoris
otu180903	-26.214	1.25E-03	g__Dorea	s__Dorea_formicigenerans
otu124251	-10.693	2.35E-03	g__Chloroplast	s__Punica_granatum
otu456393	-9.926	2.58E-03	g__Lactobacillus	s__Lactobacillus_acidophilus
otu388266	-24.376	2.72E-03	g__Bacteroides	s__Bacteroides_ovatus
otu438800	-16.207	1.25E-02	g__Prevotella	s__Prevotellaceae_bacterium
otu378363	-20.574	1.34E-02	g__Bacteroides	s__Bacteroides_ovatus
otu869840	-19.578	2.12E-02	g__Alloprevotella	s__Prevotellaceae_bacterium
